# Evaluation of bromadiolone combined with ciprofloxacin, vitamin D, aspirin, and cinnamon as an apoptosis-mediated rodenticide strategy

**DOI:** 10.1038/s41598-025-28468-7

**Published:** 2025-12-08

**Authors:** Heba Allah Ahmed Mahmoud Ahmed, Magdy Wilson, Alshaimaa A. I. Alghriany, Mona M. Atia

**Affiliations:** 1 Plant Protection Research Institute (PPRI), Agriculture Research center, Animal pests Department, Giza, Egypt; 2 Laboratory of Molecular Cell Biology, Department of Zoology and Entomology, Faculty of Science, Assiut University, Asyut, Egypt

**Keywords:** Bromadiolone, Ciprofloxacin, Vitamin d, Aspirin, Cinnamon, DNA damage, Biochemistry, Chemical biology, Drug discovery, Medical research

## Abstract

This manuscript explores whether co-formulation of the second-generation anticoagulant bromadiolone with additives such as ciprofloxacin, vitamin D, aspirin, and cinnamon can enhance rodenticidal efficacy at reduced doses, while assessing hepatic pathomorphology, oxidative stress, coagulation, DNA damage, and apoptosis in wild rats. The study is both timely and relevant, addressing ecological and public health concerns by investigating mechanistic pathways including the p53–p21–caspase axis, lipid peroxidation, comet assay, prothrombin time, and serum enzyme levels. A key strength lies in its novel strategy of combining bromadiolone with pharmacological and food-derived compounds, offering practical implications for minimizing environmental impact. The multidimensional dataset spanning biochemical, molecular, genotoxic, coagulation, and histopathological endpoints provides strong mechanistic depth. Findings suggest that certain additives, particularly cinnamon and aspirin, potentiate oxidative stress and apoptosis, correlating with increased mortality and liver damage. Overall, the integration with existing literature on vitamin K antagonism, cholecalciferol-induced hypercalcemia, NSAID-mediated apoptosis, and coumarin derivatives highlights the study’s mechanistic grounding and translational relevance.

**Supplementary Information:**

The online version contains supplementary material available at 10.1038/s41598-025-28468-7.

## Introduction

Several issues related to specific rodent species affect crop protection, human health protection, and environmental preservation. These issues pose unacceptable levels of danger and cost to the community. To mitigate the detrimental impacts of rats in an efficient, economical, ethical, and environmentally safe manner, effective techniques are needed^1,2^. Rodents can destroy almost any crop cultivated globally, including cotton, sugarcane, alfalfa, cereal grains, potatoes, vegetables, tree fruits, and many others^3^. In addition, rodents are responsible for spreading a variety of human and animal diseases, either directly or indirectly^4^. The most practical and economically feasible method of controlling rodents in agricultural fields is the chemical control of rodent pests^5^. Anticoagulants and non-anticoagulants are the two primary types of rodenticides, each with distinct rates of action (acute, subacute, and chronic)^6,7^. One common superwarfarin is bromadiolone (BDL), a long-acting dicoumarin rodenticide of the second generation. Unfortunately, as the use of this drug increased, so did the number of poisoning incidents. Superwarfarin poisoning is a global health issue^8^. Chronic diseases can arise as a result of oxidative stress, which occurs when free radicals outweigh the body’s antioxidant defenses, leading to oxidative damage to proteins and lipids^9^. Anticoagulant rodenticides are anti-vitamin K compounds that produce similar effects in mammals. Furthermore, this may eventually impact the physiological processes of higher vertebrates^10^.

Anticoagulant rodenticide poisoning is the most prevalent vitamin K-responsive coagulopathy^11^. Laboratory tests have demonstrated the effectiveness of a combined bait formulation of bromadiolone and cholecalciferol against key rodent species, with notable mortality occurring at significantly lower and over dosages^12,13^. Cholecalciferol is one of five types of vitamin D, Excessive vitamin D intake can elevate blood calcium levels, resulting in symptoms such as renal damage, nausea, vomiting, constipation, and abdominal pain. Nephrocalcinosis and kidney stones may arise from increased calcium excretion in the urine^14^. Cardiovascular risks^15^, as well as coma, insanity, and confusion, can result from severe poisoning^16,17^. Coumarin, a chemical component found in foods such as cinnamon, can inhibit vitamin K epoxide reductase complex 1, the enzyme responsible for recycling vitamin K ^18,19^. Acetylsalicylic acid (ASA), commonly known as aspirin, is used to reduce fever, inflammation, and pain, as well as to prevent thrombosis by impairing platelet function^20^. Albino rats given repeated doses (1 g/kg) of acetylsalicylic acid exhibited varying degrees of gastroenteritis, hepatitis, nephritis, pulmonary edema, and a slightly increased risk of hemorrhagic stroke^20,21^. Ciprofloxacin, a second-generation fluoroquinolone antibiotic, is considered the most effective quinolone^22^.

It is frequently prescribed for prostatitis, orchitis, and urinary tract infections; however, gonadal toxicity in humans has been reported. A common side effect of warfarin treatment is an increased risk of severe bleeding, particularly when used concurrently with drugs that affect its metabolism^23^. Specifically, interactions between various antibiotics may counteract the anticoagulant action of warfarin through multiple mechanisms^24^.

To date, no research has systematically investigated the combined effects of bromadiolone with commonly used pharmacological and dietary additives on organ pathology, DNA damage, and apoptosis. This study uniquely explores how ciprofloxacin, vitamin D, aspirin, and cinnamon influence the toxicity profile of bromadiolone in wild rats, with a particular focus on oxidative stress, antioxidant defense, and apoptotic signaling pathways. By evaluating these combinations, the work not only provides novel mechanistic insights into rodenticide-induced liver alterations but also introduces a potentially sustainable approach to rodent control. The strategy of enhancing bromadiolone efficacy with specific additives holds promise for reducing the required dose of the rodenticide, thereby lowering environmental burden and minimizing risks to non-target organisms—an aspect that has not been addressed in previous toxicological studies.

## Materials and methods

### Chemicals

Bromadiolone (0.2%) was obtained from the Biotech Company for Fertilizers and Biopesticides, Cairo, Egypt. Ciprofloxacin, vitamin D, and acetylsalicylic acid were purchased from a commercial pharmacy. Dried cinnamon (*Cinnamomum zeylanicum*) bark was obtained from the commercial market in Assiut, Egypt. EGTA, SDS-PAGE electrophoresis chemicals, nitrocellulose membranes, protease inhibitor, mouse anti-cleaved caspase-3 (1:1000), p21, p53, goat anti-β-actin IgG, and mouse anti-goat IgG-HRP were purchased from Sigma-Aldrich (St. Louis, MO, USA). 5,5′-dithio-bis-(2-nitrobenzoic acid) was obtained from Sigma-Aldrich. GSH was determined using commercial kits (Bio-Diagnostic Company, Egypt).

### Ethical statment

We confirm that our study was conducted following ARRIVE guidelines. All experimental procedures were conducted following the guidelines of the National Institutes of Health. The medical ethics committee of the Assiut University Faculty of Science reviewed and approved the research methods **(IRB No.: 01-2025-0009).**

### Cinnamon extraction

The preparation of the cinnamon extract followed the methodology described in ^25^. In brief, 10 g of cinnamon bark were cleaned and ground into a fine powder using a mechanical grinder. The powder was soaked in 100 mL of distilled water and boiled at 100 °C for 2 h. The mixture was then filtered and dried overnight in an oven at 80 °C. The resulting dry extract was weighed and stored for administration. Approximately 20% (w/w) of the crude aqueous extract of cinnamon bark was obtained.

### Experimental design

Male wild rats of the species *Rattus norvegicus*, weighing 150–200 g, were captured alive from the wheat granary of the Agriculture Bank of Egypt in Assiut Governorate in 70 traps. They were transported in a special animal transport vehicle, and each rat was in its own trap. During the transport, each trap had a bottle of water and enough food until it reached to the animal house. They were housed individually in traps (tomatoes inside the traps) for one week in the animal facility of the Zoology Department, Faculty of Science, Assiut University, at room temperature (25 °C) under a 12-hour light/dark cycle. Each animal was provided with tap water, soggy bread, and tomatoes. The trapped rats were used to perform two experiments:

### Experiment I (determination of $\:{\mathbf{L}\mathbf{D}}_{50}$ and LT_50_ of bromadiolone)

An approximate LD₅₀ was initially determined as a pilot study using the staircase method^26^. Seventy adult male rats weighing 150–200 g were randomized and divided into ten groups (*n* = 10 × 7). Group I served as the control, while groups II, III, IV, V, VI, VII, VIII, IX, and X were treated orally with the following doses of bromadiolone, respectively: 0.3, 0.6, 0.9, 1.1, 1.2, 1.4, 1.8, 2.2, and 2.8 mg/kg b.w. The animals were observed during the first 2 h and then at 6 and 24 h for any toxic symptoms. Time to death and mortality were recorded over four days following administration. After one week, the LD₅₀ value was determined using probit analysis as described in^27^.

### Experiment II (main experiment)

Adult male rats (*n* = 7 × 9 = 63), weighing 150–200 g, were randomized and divided into seven groups. Group I served as the control. Group II received the vehicle control (0.1 ml/100 g ethanol). Group III was treated with bromadiolone (B) at ½ LD₅₀ (0.8 mg/kg)^28^. Group IV received bromadiolone (0.8 mg/kg) + ciprofloxacin (Anti.20 mg/kg)^29^. Group V received bromadiolone (0.8 mg/kg) + ciprofloxacin (20 mg/kg) + vitamin D (Vit. D 35 mg/kg)^30^. Group VI received bromadiolone (0.8 mg/kg) + ciprofloxacin (20 mg/kg) + vitamin D (35 mg/kg) + cinnamon (Cin. 2 g/kg)^31^. Group VII received bromadiolone (0.8 mg/kg) + ciprofloxacin (20 mg/kg) + vitamin D (35 mg/kg) + aspirin (A. 150 mg/kg)^32^. All doses were administered orally to wild rats over 21 days. The chemical we used for the euthanizing process: rats were narcotized by Ketamine + xylazine injection (IP) and then sacrificed by slaughtering.

### Western blot

For the immunodetection and quantification of proteins such as p21 and cleaved caspase-3, markers of apoptotic cell death under various treatments, liver tissue was homogenized using RIPA lysis buffer. The samples were chopped and briefly sonicated on ice, and protein concentration was measured. Proteins (20 µg) were separated using SDS-PAGE and transferred to a nitrocellulose membrane. Membranes were washed with 5% skim milk in TBS, then incubated with primary antibodies overnight at 4 °C, followed by incubation with HRP-conjugated secondary antibodies for 1 h at 24 °C, as recommended by the manufacturers. Immunoreactive bands were visualized using a chemiluminescent substrate kit. The optical densities of the bands were quantified and normalized to β-actin (1:5000) using ImageJ software.

### Comet assay

For the coated slides, 100 µL of cell suspension was mixed with 600 µL of low-melting agarose (0.8% in PBS). The coated slides were immersed for 15 min in a lysis solution containing 2.5% sodium dodecyl sulfate (SDS) and 0.045 M Tris-borate-EDTA (TBE) buffer at pH 8.4. The slides were then placed in an electrophoresis chamber filled with TBE buffer without SDS. Electrophoresis was conducted at 100 mA for two minutes at 2 V/cm. Staining was performed at 4 °C using 20 µg/mL ethidium bromide (EtBr). EtBr-stained DNA was visualized using a fluorescent microscope with a 40× objective and an excitation filter of 420–490 nm to detect DNA damage^33^. Qualitative and quantitative assessments of tail length, tail moment, olive tail moment, and percentage of DNA in the head were determined using OpenComet software to evaluate five images.

### Detection of P53 by immunohistochemistry

Using xylene, paraffin-embedded tissues were deparaffinized and then rehydrated through a graded series of ethanol solutions. Antigen retrieval was performed by boiling the slides in 1 mM EDTA. The sections were then incubated in 3% H_2_O_2_ for ten minutes, washed with wash buffer (1X PBS) for five minutes, and blocked for one hour at room temperature. A 1:1000 dilution of the p53 primary antibody was then applied. After removing the antibody solution, the sections were washed with wash buffer for ten minutes. Each component was incubated for 30 min before adding and removing the secondary antibodies (1:5000). The sections were washed, stained with 3,3′-diaminobenzidine (DAB), and counterstained for two to three minutes^34^.

### **Apoptosis detection by acridine orange**

Before staining with 0.01% acridine orange (AO) (stock solution: 0.1% AO in distilled water, diluted with phosphate buffer to a staining solution at pH 7.2), the sample was quickly passed through a graded alcohol series (80 − 50%) followed by distilled water. The sample was immediately washed with phosphate buffer, placed in PBS for one minute, differentiated for two minutes in 0.10 M CaCl_2_, and then mounted wet with a cover glass for analysis^35^. When exposed to blue light, the monomeric form of acridine orange exhibits sharp green fluorescence under a microscope at 488 nm. Acridine orange emits sharp green fluorescence when bound to DNA (apoptosis).

### Serum measurements

Determination of ionized calcium, RBC count, Hb, and prothrombin time (PT) was conducted at a private biochemical laboratory in Assiut, Egypt, using a Genuri KT-6400 automatic hematology analyzer (China). ALT and AST activities in blood plasma were measured using specific kits (Boehringer Mannheim, Mannheim, Germany) according to the manufacturer’s instructions. Liver lipid peroxidation (LPO) was quantified using the thiobarbituric acid reaction^36^. The concentration of reduced glutathione (GSH) was estimated based on^37^ and measured using spectrophotometry.

### Examination of histopathological and histological lesions

Pieces of liver tissue were stored in 10% neutral formalin (pH 7.2) for histological examination. Hematoxylin, eosin stains was applied to 5 μm-thick paraffin Sect^38^. Four categories were established based on the histological evaluation of nine liver tissue lesions: (−) no lesion, (+) mild (less than 25%), (++) moderate (between 25% and 50%), and (+++) severe (more than 50%)^39^. We examined about 5 photos for each rat in each groups; 5 × 63 = 315 photos for assessments. Examination and photography were conducted using a digital camera (ToupTek ToupView, copyright 2019, version x86, compatible with Windows XP/Vista/7/8/10, China) and a light microscope (Olympus CX31, Japan).

### Statistical analysis

Statistical analysis was conducted using analysis of variance. Data were presented as the mean ± S.E. from at least three independent experiments, and differences were considered significant at *P* < 0.05 and *P* < 0.0001. One-way analysis of variance (ANOVA) and Student’s t-test were used. Fiji/ImageJ and OpenComet were used for western blot and graph image analysis. SPSS, Prism software version 8, and Probit were used for data processing.

## Results

### Determination of $\:{\mathbf{L}\mathbf{D}}_{50}$ and $\:{\mathbf{L}\mathbf{T}}_{50}$

**Fig. 1:**
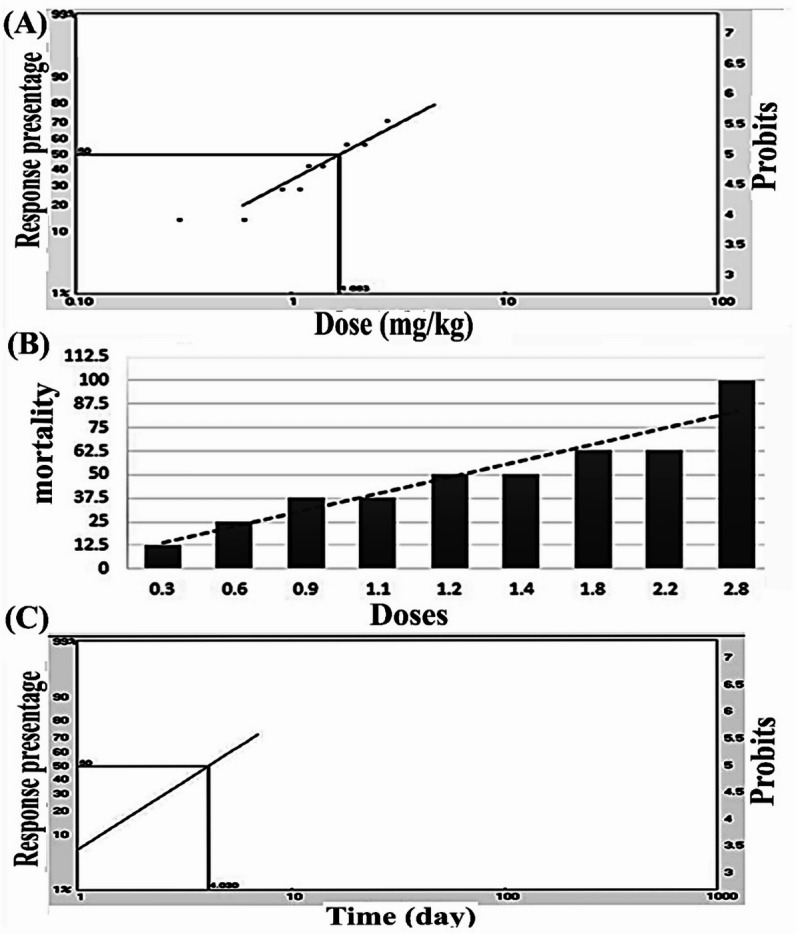
(**A**) Plot of doses (mg/kg b.w.) versus probits from Table 2 for the calculation of LD₅₀ of bromadiolone administered orally. (B) Acute oral toxicity LD₅₀ of different doses of bromadiolone against male wild rats *Rattus norvegicus* based on probit analysis and SPSS statistics. **(C)** Plot of time (days) for calculation of $\:{\mathbf{L}\mathbf{T}}_{50}\:$of Bromadiolone administered orally.

All groups were administered different doses of bromadiolone orally (0.3, 0.6, 0.9, 1.1, 1.4, 1.8, 2.2, and 2.8 mg/kg body weight) to estimate the LD₅₀. After treatment with the aforementioned doses for one week, mortality was recorded in Table 1, and 2, as well as in (Figs. 1A and B). The analysis yielded an LD₅₀ of 1.6 mg/kg. The time required to achieve total mortality ranged from 4.0 to 6.0 days. Moreover, in our work, based on the previous doses, the results of the Probit analysis revealed that the value of LT₅₀ for males ranged from 2.187 to 4.03 days, as shown in Tables 3 and 4 and (Fig. 1C).

**Table 1:** Results from probit analysis of the lethal doses of Bromadiolone for the determination of $\:{\mathbf{L}\mathbf{D}}_{50}$ after oral administration of male wild rats of *Rattus norvegicus*.

Dose	Dose*10	Log (Dose*10)	Treated	Dead	Mortality%	Observed response %	Linear response %	Linear probit
0.3	3	0.4771	8	1	12.5	14.286	8.15909	3.6051
0.6	6	0.7782	8	2	25	14.286	20.3258	4.1699
0.9	9	0.9542	8	3	37.5	28.571	30.855	4.5
1.1	11	1.0414	8	3	37.5	28.571	36.8278	4.6635
1.2	12	1.0792	8	4	50	42.857	39.5276	4.7344
1.4	14	1.1461	8	4	50	42.857	44.4304	4.8599
1.8	18	1.2553	8	5	62.5	57.143	52.578	5.0647
2.2	22	1.3424	8	5	62.5	57.143	59.022	5.2281
2.8	28	1.4472	8	8	100	71.429	66.4427	5.4246

**Table 2:** Results of the lethal doses of Bromadiolone for the determination of $\:{\text{L}\text{D}}_{25}$ to $\:{\text{L}\text{D}}_{99}$ by probit analysis software.

LD	Dose (mg/kg)
25	0.7264
50	1.6626
75	3.8056
90	8.0187
95	12.5257
99	28.9136

**Table 3:** Results from probit analysis of the time lethal doses of Bromadiolone for the determination of $\:{\mathbf{L}\mathbf{T}}_{50}$ after oral administration of male wild rats of *Rattus norvegicus*.

Time	Time* 1	Log (Time*1)	Treated	Observed response %	Linear response %	Linear probit
1	1	0	8	12.5	6.20873	3.4617
2	2	0.301	8	12.5	21.9652	4.2266
3	3	0.4771	8	37.5	37.2284	4.6741
4	4	0.6021	8	50	49.6729	4.9918
5	5	0.699	8	50	59.4089	5.2381
6	6	0.7782	8	62.5	66.9788	5.4393
7	7	0.8451	8	87.5	72.8843	5.6093

**Table 4:** Results of the lethal time of Bromadiolone for the determination of $\:{\text{L}\text{T}}_{25}$to $\:{\text{L}\text{T}}_{99}$ by probit analysis software.

LT	Time (day)	Lower limit day	Upper limit day
25	2.187	0.822	3.091
50	4.03	2.771	6.273
75	7.426	5.129	23.18
90	12.87	7.592	88.39
95	17.89	9.453	200
99	33.17	14.11	934.7

### Pathomorphological symptoms of Bromadiolone (LD_50_) in male wild rats

**Fig. 2:**
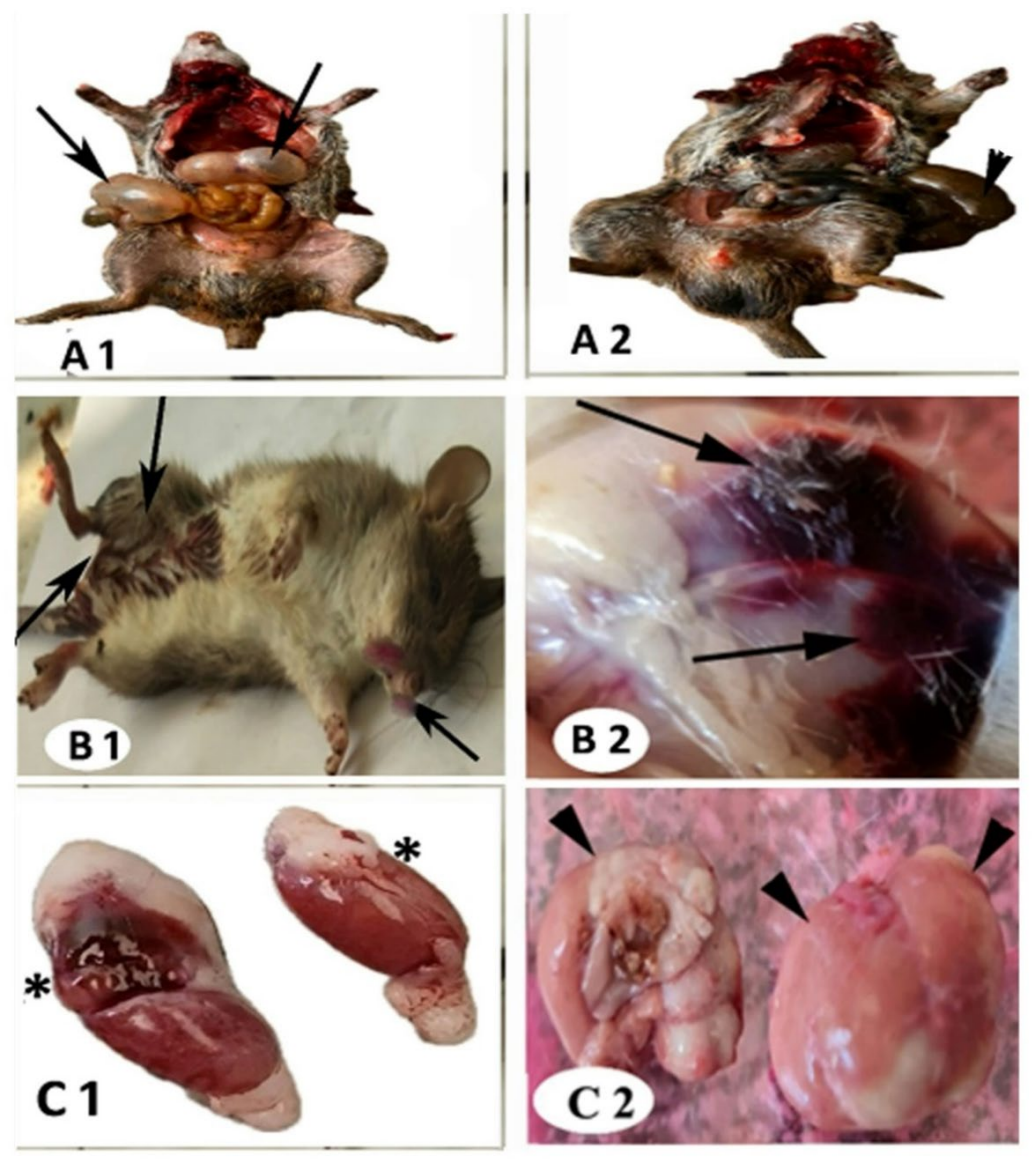
Pathomorphological symptoms of male wild rats treated with different doses of bromadiolone, showing changes in physical appearance and internal organs (liver and testis): (A1) Abnormal appearance, enlargement, and severe bloating of the digestive tract organs (black arrow). (A2) Bleeding into body cavities, congestion, and shrinkage of the liver and intestine (black arrowhead). (B1) Enlarged head, bleeding near the genital openings, the penis entrance, and the mouth. (B2) Severe congestion throughout the abdomen (black arrow). (C1) Bloody and swollen appearance of testis (*). (C2) Gross pathological lesions in the liver observed during external examination show a single, well-defined mass with multiple small calcifications separated from the surrounding liver parenchyma (black arrowhead).

The toxicity symptoms of bromadiolone poisoning began to appear 2–4 days after oral administration and worsened after 7 days. Dormancy, emaciation, breathing difficulties, and hemorrhages in various areas, including the abdomen (Figs. 2A1 and A2), the penile opening, testis (Figs. 2B1 and B2), nose, eyes, mouth, fingertips, back, femur, and tail (Figs. 2C1 and C2), were observed. Widespread wounds, bleeding into body cavities, and the presence of blood in urine or feces represent specific clinical signs (Figs. 2D1 and D2). Severe and sudden bleeding may lead to cardiovascular shock and death.

### Western blot analysis for P21 and cleaved caspase-3

**Fig. 3:**
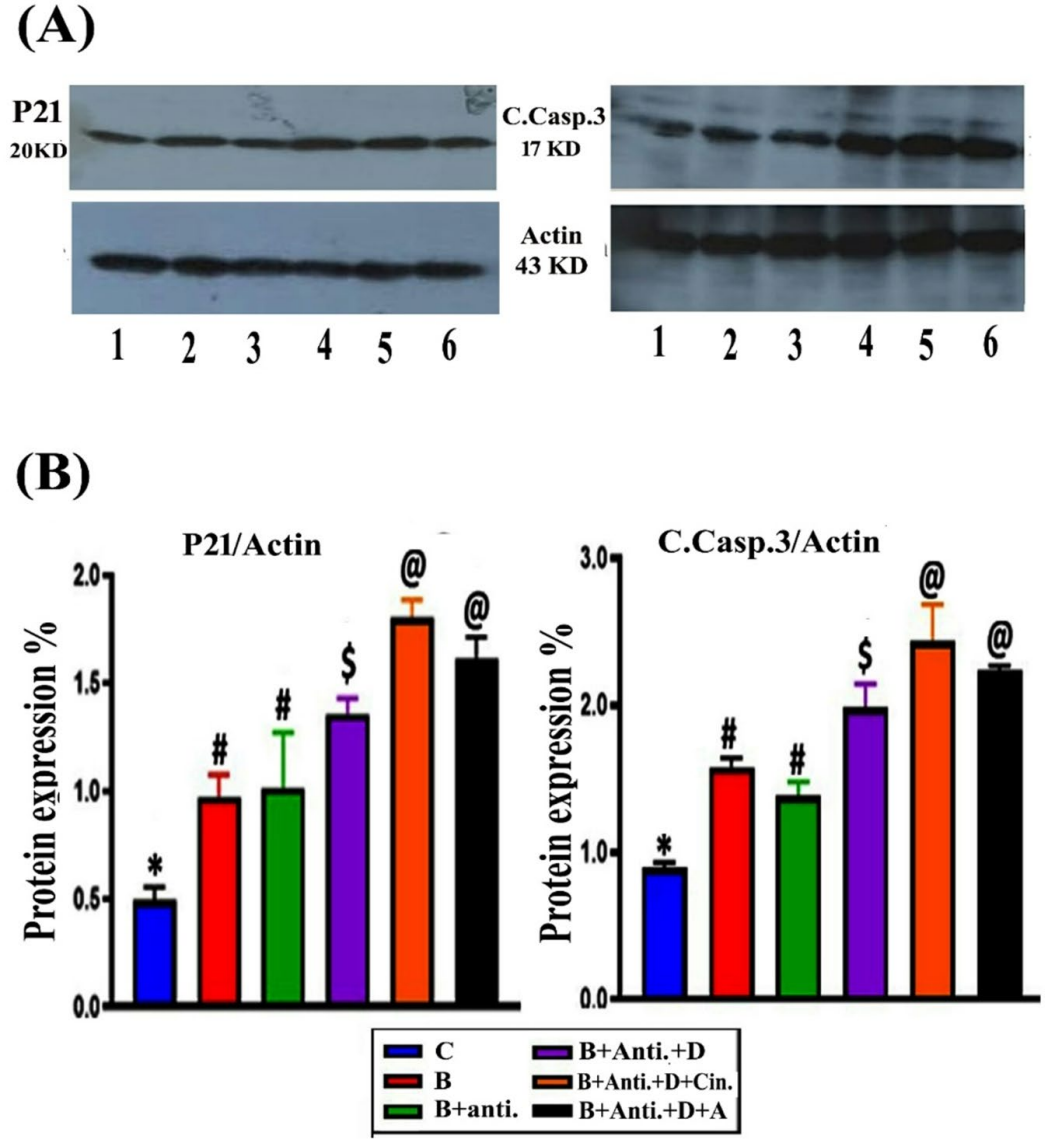
Western blot analysis: (**A**) Detection of P21 and cleaved caspase-3 levels in liver sections from different treatment groups (1) C; (2) B; (3) B + Ant.;(4) B + Ant.+ D; (5) B + Ant.+ D + Cin. (6) B + Ant.+ D + A. (B). (**B**) Percentage induction of protein/actin ratio after normalization to the control level, with significantly different symbols marked on the columns (full image of the blots in supplementary material).

Changes in protein levels of p21 and cleaved caspase-3 under various treatments were determined by Western blot analysis. The differently treated groups (B, (B + Anti), (B + Anti + vit. D), (B + Anti + vit. D + Cin), and (B + Anti + vit. D + A)) showed significant increases in p21 and cleaved caspase-3 levels by 96.65%, 105.12%, 173.98%, 264.23%, and 225.68%, and 75.92%, 54.76%, 121.94%, 172.08%, and 149.53%, respectively, compared to the control (Figs. 3A and B).

### Investigation of immunohistochemistry

**Fig. 4:**
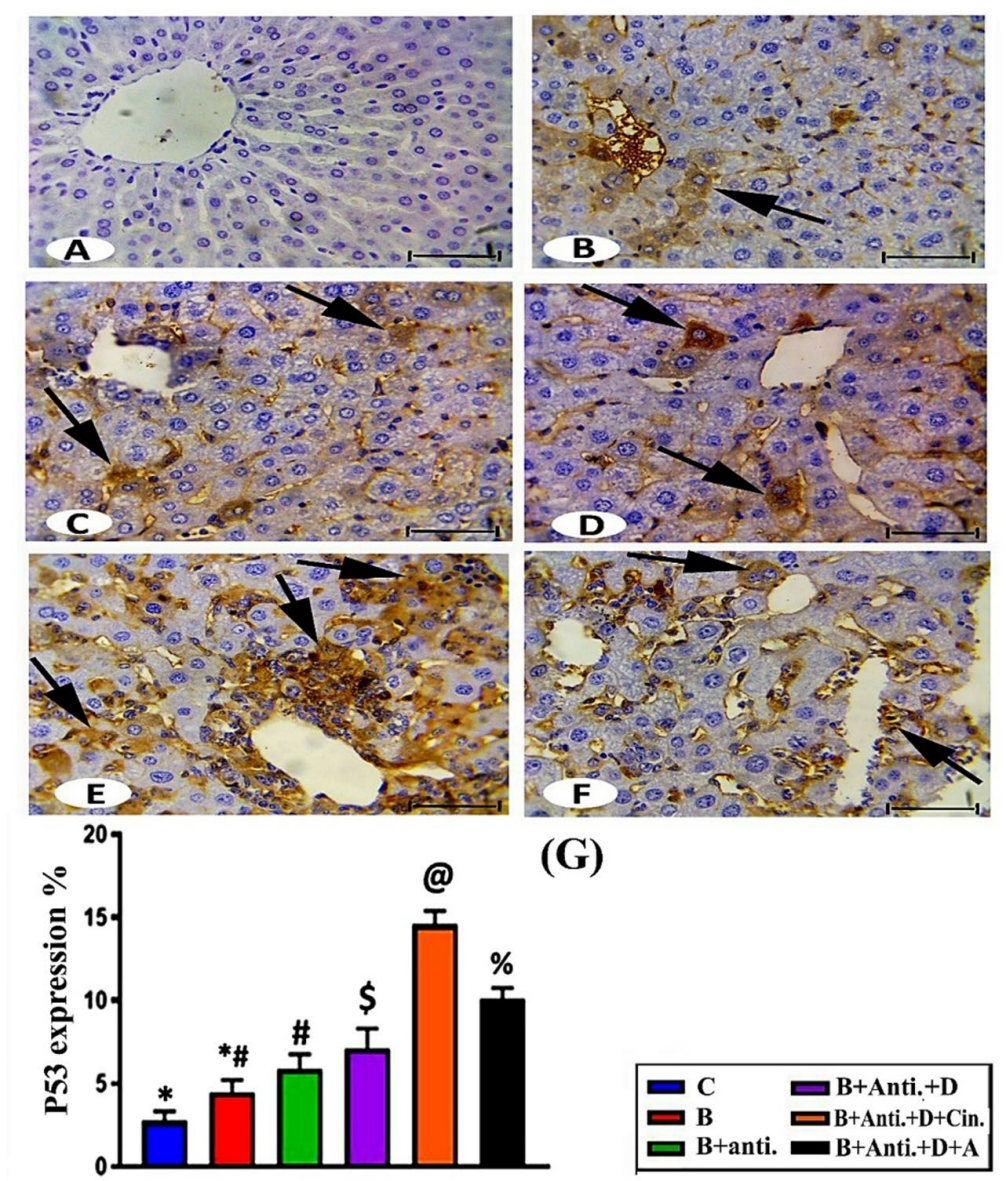
Immunohistochemical detection of pro-apoptotic effector p53 in liver sections: (**A**) Control groups (I and II) reveal a negative reaction; (**B**) Group III demonstrates moderate brown immunoreactivity of p53; (**C**,** D**,** E**, and **F**) Groups IV, V, VI, and VII show progressively increased p53 immunoreactivity in hepatic cells (arrows indicate positive reaction). Scale bar: 50 μm. (G) Statistically, the values in columns with different superscript signs were significantly different.

Immunohistochemical detection of p53 levels in the livers of control male rats showed a negative reaction or weak staining (Fig. 4A). The intensity of p53 levels in hepatocytes (cytoplasm and around the central vein) increased in the different treatment groups, demonstrating a deep brown stain and increasing percentages in the following order: (B + Anti + vit. D + Cin) ˃ (B + Anti + vit. D + A) ˃ (B + Anti + vit. D) ˃ (B + Anti) ˃ B (Figs. 4F, E, D, C, and B). The effect of bromadiolone on wild rats after oral administration was non-significant, with a 60.74% increase in p53 levels compared to the control. In contrast, the level of p53 increased significantly by 111.72%, 157%, 424.16%, and 262.69% in groups IV, V, VI, and VII, respectively, compared to the control group (Fig. 4G).

### Evaluation of the comet assay for DNA damage

**Fig. 5:**
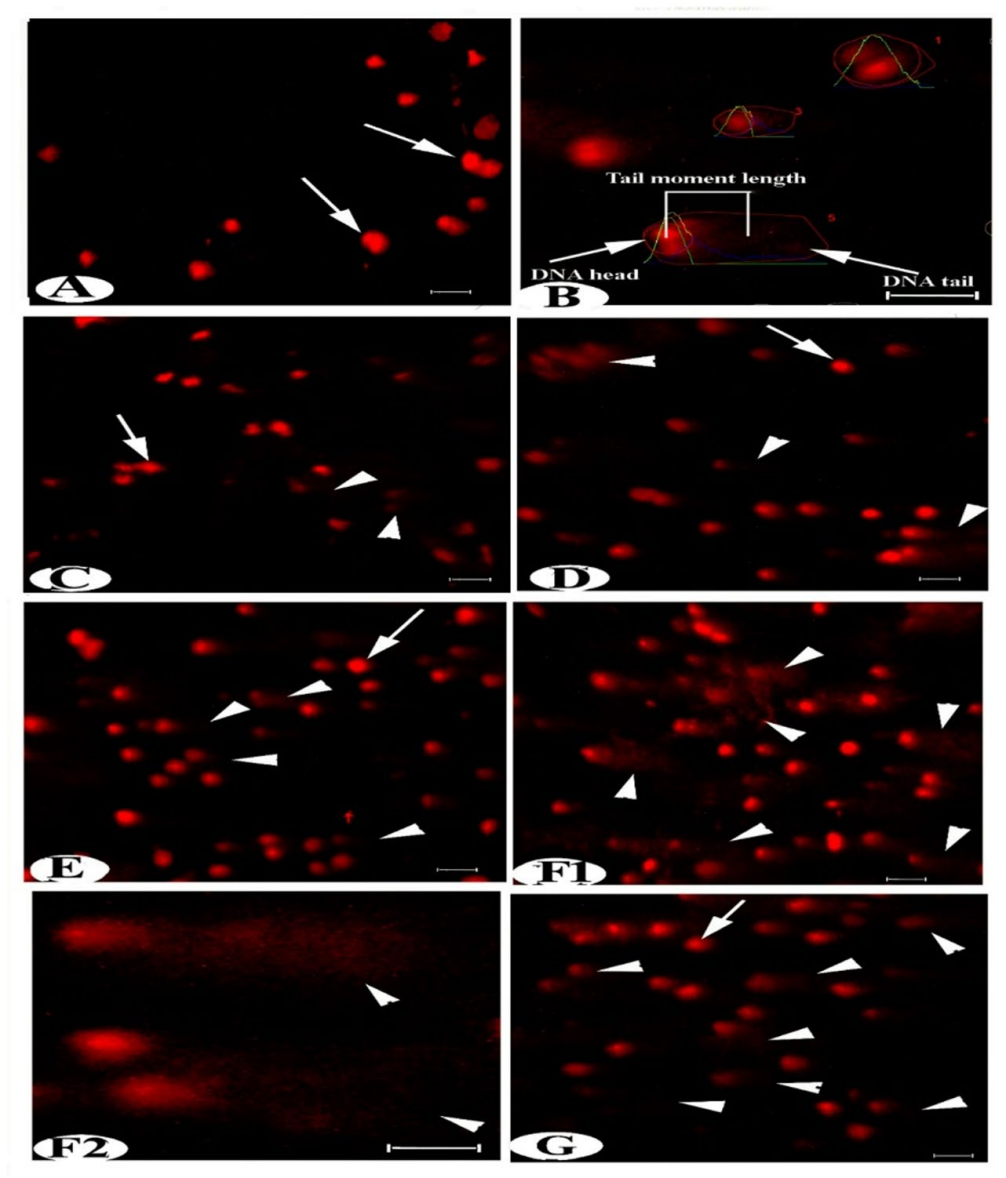
Photomicrographs of fluorescence microscope images of liver DNA norvegicus by Comet assay showing **(A)** group (I and II) normal liver with intact DNA, (arrow). **(B)** Comet assay parameters for detection of DNA damage (olive tail moment, tail moment, tail DNA precent and tail length). **(C)** group III with a moderate amount of damaged DNA (arrowhead), **(D and E)** groups IV and V show a high degree of DNA damage. **Scale bar: 100 μm**. **(F1 & F2)** group VI shows severe and acute damaged DNA with numerous comets (arrowhead), scale bar 100 & 50 μm, respectively. (**G**) Group VII shows a degree of DNA damage.

**Fig. 6:**
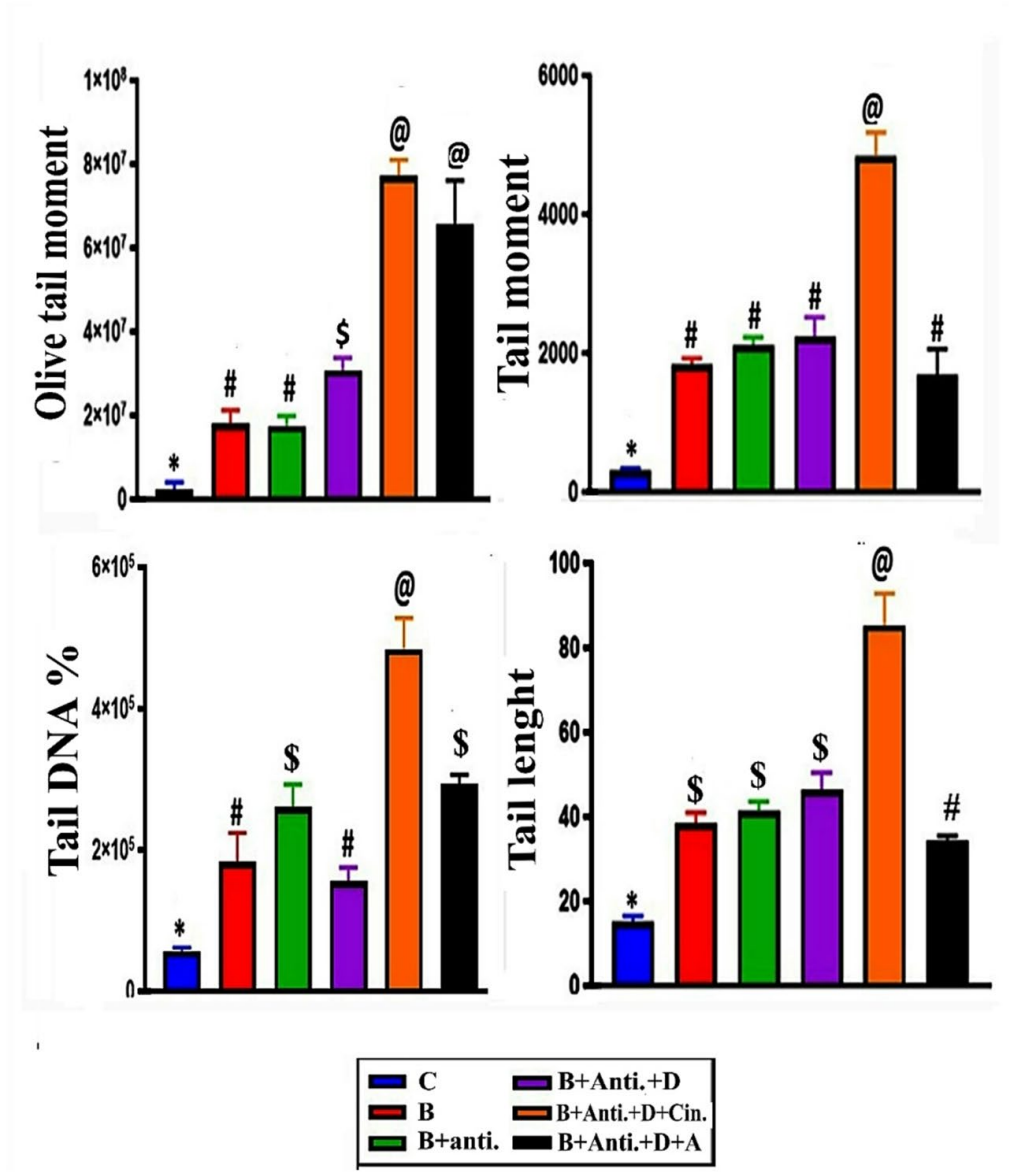
Diagrams show the effect of experimental treatment groups on comet assay analysis (olive tail moment, tail moment, tail DNA percentage, and tail length), with statistically significant differences (*p* < 0.0001 and 0.005) represented by data with different superscript signs.

Olive tail moment (OTM), tail moment (TM), tail DNA% (T.DNA% %), and tail length (TL) parameters were analyzed for each comet on each slide, and the average values were used to determine DNA damage in the liver of male wild rats *Rattus norvegicus*. OTM, TM, T.DNA%, and TL data in all treated groups (B, (B + Anti), (B + Anti + vit. D), (B + Anti + vit. D + Cin), and (B + Anti + vit. D + A) showed highly significant increases of 710.02%, 685.14%, 1285.79%, 3383.08%, and 2861.85% in OTM; 506.09%, 596.38%, 635.26%, 1490.61%, and 456.01% in TM; 222.35%, 360.47%, 176.02%, 756.17%, and 418.37% in T.DNA%; and 155.04%, 173.81%, 208.16%, 466.25%, and 128.38% in TL, respectively, particularly in the cinnamon group compared to the control (Figs. 5 and 6).

### Apoptosis estimation by AO fluorescence staining

**Fig. 7:**
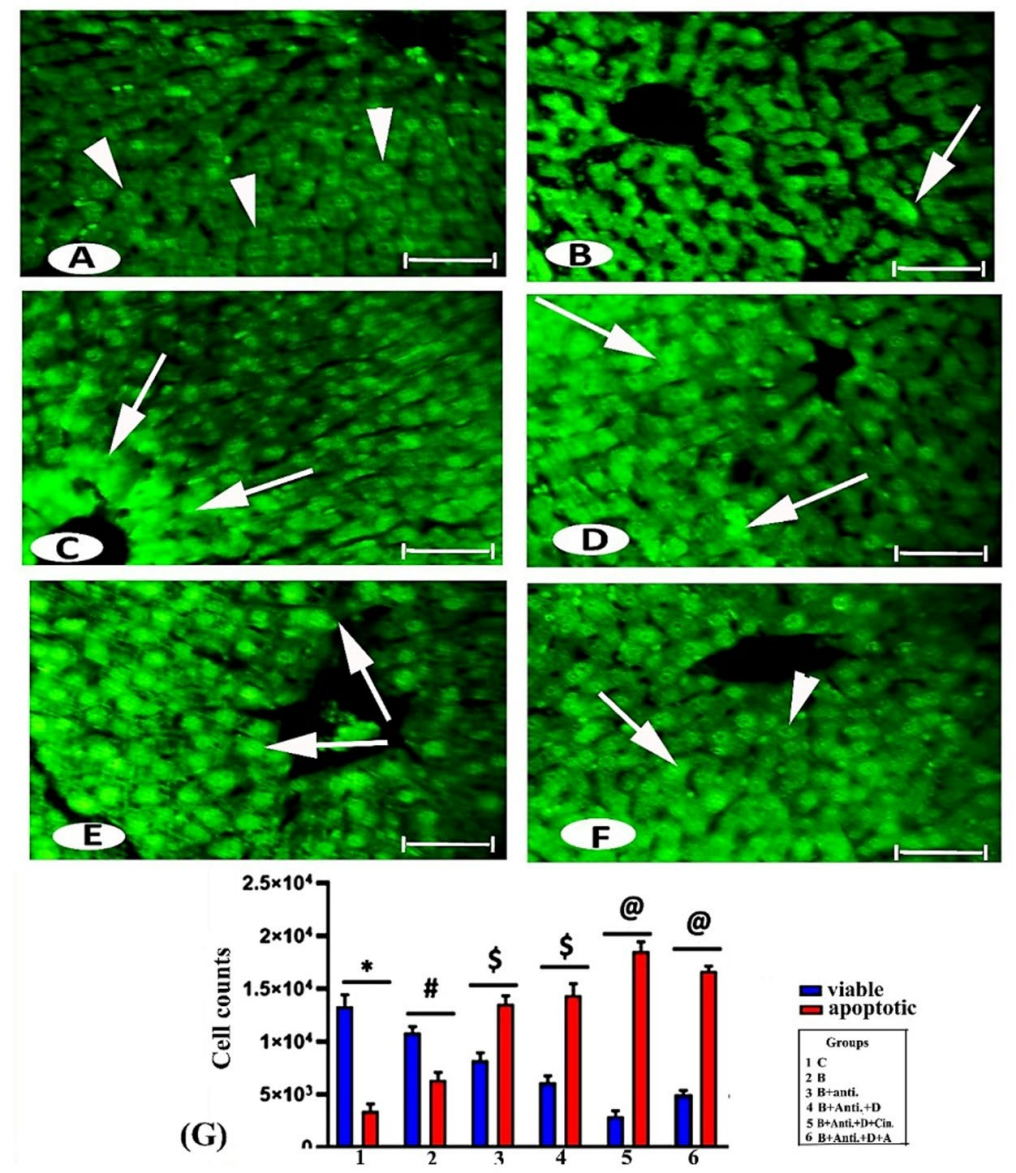
Photomicrographs from fluorescence microscope images of liver cells in the experimental groups: (**A**) Control; (**B**) Bromadiolone; (**C**) B + Anti; (**D**) B + Anti + Vit. D; (**E**) B + Anti + Vit. D + Cin; and (**F**) B + Anti + Vit. D + A, stained with AO dye. Normal cells show faint green nuclei (head arrow), while pyknotic nuclei appear with bright green fluorescence (arrow). Scale bar: 50 μm. (**G**) Statistically, the values in columns with different superscript signs were significantly different.

To study apoptosis induction, we observed changes in the nucleus and chromatin material under a fluorescence microscope after staining with AO. Live cells appeared uniformly faint green, while early apoptotic cells were stained with bright fluorescent green dots inside the nuclei due to chromatin condensation and nuclear fragmentation. No significant apoptosis was detected in the control and ethanol control groups, which exhibited few dead cells and numerous normal cells (Fig. 7A).

The treated groups (B, (B + Anti), (B + Anti + vit. D), (B + Anti + vit. D + Cin), and (B + Anti + vit. D + A)) showed a significant decrease in normal cells by 18.66%, 38.34%, 54.05%, 77.63%, and 62.6%, respectively, and a significant increase in apoptotic cells by 109.72%, 287.93%, 311.59%, 431.93%, and 377.03%, respectively, compared to the control. All treated groups exhibited morphonuclear changes such as pyknotic nuclei (Figs. 7B, C, and G) and dense, brightly green-stained chromatin, indicating late-stage apoptosis. Highly intense and asymmetrically localized green nuclear staining was observed with increasing concentrations of additive substances, particularly with the addition of cinnamon (Figs. 7D, E, and F).

### Biochemical analysis

#### Selected biochemical parameters in blood serum

AST, ALT, and calcium levels significantly increased in all treated groups (B, (B + Anti), (B + Anti + vit. D), (B + Anti + vit. D + Cin), and (B + Anti + vit. D + A)) compared to the control, with increases of 41.55%, 78.71%, 101.86%, 168.56%, and 127.31% in AST; non-significant and significant increases of 22.02%, 16.51%, 36.22%, 97.08%, and 108.39% in ALT; and 183.41%, 140.95%, 268.97%, 402.59%, and 304.31% in calcium, respectively.

Nevertheless, RBC and Hgb counts significantly decreased by 25.63%, 26.08%, 44.95%, 60.29%, and 54.33%, and by 15.94%, 33.05%, 48.32%, and 30.70%, respectively. PT levels significantly increased by 76.14%, 338.49%, 578.82%, 1116.71%, and 1047.06%, respectively (Table 5).

**Table 5:** Estimation of serum of AST, ALT, Ca^2+^, RBCs, Hgb and PT levels and hepatic LPO and GSH, in different experimental groups of male wild rats.

	Male Rattus norvegicus										
	Control	B		(B + anti)		(B + anti + vit. D)		(B + anti + vit. D + C)		(B + anti + vit. D + A)	
	Mean ± SE	Mean ± SE	% of change	Mean ± SE	% of change	Mean ± SE	% of change	Mean ± SE	% of change	Mean ± SE	% of change
AST (U/L) serum	92.37 ± 5.279 *	130.8 ± 8.149 **#**	41.55% ↑	165.1 ± 5.088 **$**	78.71% ↑	186.5 ± 3.686 **$%**	101.86% ↑	248.0 ± 4.954 **@**	168.56% ↑	210.0 ± 9.578 **&**	127.31% ↑
ALT (U/L) serum	184.8 ± 4.632 *	225.5 ± 1.679 *	22.02% ↑	215.3 ± 1.715 *	16.51% ↑	251.7 ± 7.382 *	36.22% ↑	364.2 ± 38.45 **@**	97.08% ↑	385.1 ± 18.23 **@**	108.39% ↑
LPO (nmol/ mg protein)	1.458 ± 0.1402 *	1.660 ± 0.1817*	13.69% ↑	2.530 ± 0.2937**#**	73.29% ↑	2.984 ± 0.2613**#**	104.79% ↑	5.313 ± 0.3714**@**	264.38% ↑	3.903 ± 0.2003 **$**	167.12% ↑
GSH (ng/mg protein)	10.42 ± 0.4500 *	6.953 ± 0.1538**#**	33.21% ↓	5.893 ± 0.2378**#**	43.38% ↓	5.177 ± 0.7614#	50.29% ↓	3.823 ± 0.4656**@**	63.24% ↓	5.373 ± 0.3210 **#**	48.37% ↓
Ca2+ (mg/dl) serum	4.640 ± 0.2943 *	13.15 ± 0.9675 #	183.41% ↑	11.18 ± 0.8499 #	140.95% ↑	17.12 ± 1.965 $	268.97% ↑	23.32 ± 2.970 @	402.59% ↑	18.76 ± 2.705 $	304.31% ↑
RBCs (M/µl) serum	11.08 ± 0.1956 *	8.240 ± 0.5015 #	25.63% ↓	8.192 ± 0.2049 #	26.08% ↓	6.098 ± 0.9436 $	44.95% ↓	4.396 ± 0.8644 @	60.29% ↓	5.054 ± 0.9225 $	54.33% ↓
Hgb (g/dl) serum	11.92 ± 0.3813*	11.4 ± 0.2358*	7.38% ↓	10.02 ± 0.8279 #	15.94% ↓	7.980 ± 1.403 $	33.05%	6.160 ± 1.145 @	48.32%	8.260 ± 1.047 $	30.70% ↓
PT (Sec.) serum	21.25 ± 2.212 *	37.43 ± 1.329 *	76.14% ↑	93.18± 3.277 $	338.49% ↑	144.2 ± 7.471 #	578.82% ↑	258.6 ± 8.295 @	1116.71% ↑	243.8 ± 9.290 @	1047.06% ↑

Data is represented as mean ± SE, statistically significant difference (*p* < 0.0001, and 0.005) were represented by data with different signs.

#### Hepatic antioxidant (GSH) and oxidative stress (LPO) parameters

GSH levels significantly decreased by 33.21%, 43.38%, 50.29%, 63.24%, and 48.37% in all treatment groups (B, (B + Anti), (B + Anti + vit. D), (B + Anti + vit. D + C), and (B + Anti + vit. D + A), respectively. In contrast, LPO levels showed a non-significant increase of 13.69% and significant increases of 73.29%, 104.79%, 264.38%, and 167.12%, respectively, compared to the control (Table 6).

**Table 6:** Semi-quantitative scoring of histopathological lesions in the liver of the examined groups.

Lesions	Groups					
	Control	B	B + Anti	B + Anti + D	B + Anti + D + C	B + Anti + D + C
Loss of liver architecture	**-**	**-**	**+**	**++**	**+++**	**+++**
**Features of necrosis:**						
- Pyknosis	**+**	**++**	**++**	**+++**	**+++**	**+++**
- Karyorrhexis	**-**	**+**	**+**	**+**	**+**	**++**
- Karyolysis	**-**	**+**	**++**	**+**	**++**	**++**
Vacuolated cytoplasm	**+**	**++**	**+++**	**+++**	**+++**	**+++**
Fatty deposition	**-**	**-**	**++**	**+++**	**+++**	**++**
Congested blood vessels	**-**	**++**	**++**	**++**	**+++**	**++**
Dilated blood sinusoids	**-**	**+**	**+**	**+**	**++**	**++**
Hemorrhage	**-**	**-**	**+**	**++**	**++**	**+++**
Thickening of blood vessels	**-**	**-**	**-**	**+**	**++**	**+**
Cellular infiltration	**-**	**++**	**++**	**+++**	**+++**	**++**
Necrotic areas	**-**	**+**	**+**	**+**	**+++**	**+++**

(-) Absent lesion, (+) Slight (< 25%), (++) Moderate (from 25–50%), and (+++) Severe (> 50%).

#### Histological and histopathological analysis

**Fig. 8:**
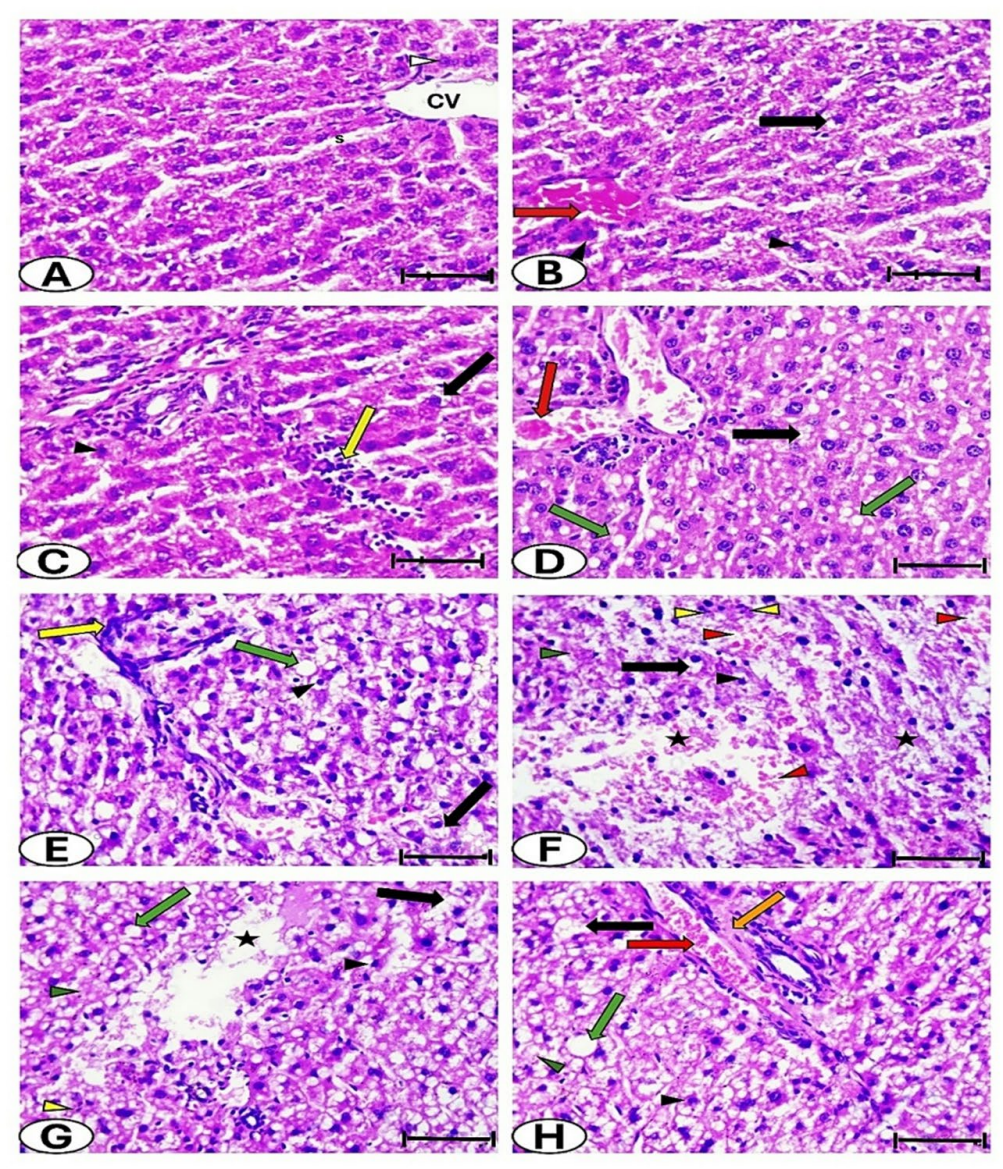
Photomicrographs of liver sections stained with H&E, scale bar = 50 μm: (**A**) Control group; (**B** & **C**) B group; (**D**) B + Anti group; (**E**) B + Anti + D group; (**F** & **G**) B + Anti + D + Cin group; and (**H**) B + Anti + D + A group. CV: central vein; S: white arrowhead, rounded vesicular nucleus; red arrow, congestion; black arrow, vacuolated cytoplasm; black arrowhead, pyknotic nucleus; yellow arrow, cellular infiltration; green arrow, fatty deposition; orange arrow, thickening around blood vessels; red arrowhead, hemorrhage; yellow arrowhead, karyorrhexis; green arrowhead, karyolysis; and black asterisk, necrotic area.

Sections of liver from the control group stained with hematoxylin and eosin revealed a nearly normal structure (Fig. 8). In group B (Figs. 8B and C), hepatocytes appeared condensed with vacuolated cytoplasm. The central vein was congested, and cellular infiltration was observed. Hepatic tissue damage progressively increased from the (B + Anti) group to the (B + Anti + D + A) group (Figs. 8D-H), in addition to the alterations seen in group B. This deterioration was characterized by loss of liver architecture, fatty deposition, hemorrhage, thickening around blood vessels, necrotic areas, and features of necrosis, including pyknosis, karyorrhexis, and karyolysis. The histopathological lesions in the livers of the studied groups were semi-quantified as presented in Table 6.

## Discussion

Around the world, anticoagulant poisons have been used for a long time to control rodents. A new threat to human life is represented by superwarfarins such as bromadiolone and brodifacoum. Following broad dispersal, the increasing use of these chemicals has raised the risk of accidental exposure to residual substances in the environment or ingestion of bait^8^. Our current study aligns with previous research indicating that natural rodent repellents are superior to synthetic ones due to their higher repellent activity and fewer adverse effects on the environment and human health^2^. We found that oral intake of bromadiolone with natural and synthetic additives, such as ciprofloxacin (antibiotic); cholecalciferol (Vit. D); natural coumarin anticoagulant (cinnamon); and the pharmaceutical anticoagulant acetylsalicylic acid (aspirin), resulted in the stimulation of ROS and oxidative stress in male wild rats. Our study examined pathomorphological changes and the median lethal time following the determination of the LD₅₀ of bromadiolone, along with gross pathological lesions in the liver. Following other studies, bromadiolone induced toxicity and mortality in rats^40^. Using a cereal bait formulation containing cholecalciferol (0.04%) and bromadiolone (0.0025%) can aid in controlling rodent populations in crops^1^. Our results indicate that combining bromadiolone with ciprofloxacin, vitamin D, or cinnamon — and, to some extent, with aspirin — enhanced markers of oxidative stress and apoptosis, suggesting the potential to achieve rodenticidal effects at lower bromadiolone doses. However, safety and ecological impacts require further evaluation.

The current findings indicate that the addition of different additives to bromadiolone-treated groups resulted in a significant increase in apoptotic proteins such as P21 and cleaved caspase-3 levels, particularly after the addition of cinnamon, as well as the appearance of dense, bright green chromatin in AO fluorescent staining, indicating DNA damage, when compared to other groups. Many signaling pathways converge at P21 to execute cell death. When active, chromatin condensation occurs, membrane proteins are damaged, and DNA degrades. Upon sustaining DNA damage, p53 accumulates in the nucleus and mitochondria, leading to the transcriptional activation of its target genes (mdm2, p21, bax, and puma). Cell death initiation and cell cycle arrest at the G1/S boundary subsequently occur^41,42^.

Recently, esculetin (hydroxycoumarin) has been shown to suppress Sp1 (specificity protein 1), a transcription factor that leads to nuclear shrinkage and fragmentation, which are common indicators of apoptosis. Notably, esculetin activated apoptotic signaling molecules such as caspase-3 and PARP in G361 HMM cells by modulating Sp1 downstream target genes, including p27, p21, and cyclin D1^43^. According to our results, a rise in p53 promotes aspirin-induced liver damage or the combined effect of bromadiolone, ciprofloxacin, Vit. D, and cinnamon. Furthermore, the upregulation of p53 led to substantial epigenetic modifications, as well as its downstream target gene p21, and liver injury. Despite this, our research indicates that p53 functions as a transcriptional regulator of apoptosis in pathological processes. Our data suggest that the concurrent increase in ROS, particularly oxidative stress (LPO), activated the p53-p21 signaling pathway, which in turn caused DNA damage due to the overexpression of p21, leading to p53-dependent G1 phase arrest^44,45^.

Animals are frequently subjected to low-level stresses, such as oxidative stress, endoplasmic reticulum stress, and inflammation, among other constitutive stresses^46^, which can induce varying degrees of p53 activity, in addition to acute stresses that cause strong p53 activation. These findings, along with our results, underscore the important role of high p53 expression and Ca level in the death of wild rats. According to several studies, serum calcium levels begin to rise approximately 24 h after cholecalciferol poisoning^12^. Similar increases in blood calcium levels in *Rattus rattus* following treatment with a standard dose of cholecalciferol have been documented by ^12^. This uncontrolled rise in serum calcium levels (hypercalcemia) may result in metabolic calcification of vital organs such as the kidneys, gastrointestinal tract, and cardiac muscle, ultimately leading to death^13^.

Apoptotic features observed through light microscopy and acridine orange (AO) staining such as cell rounding, shrinkage, and membrane blebbing along with DNA strand breaks detected via the comet assay, collectively confirmed apoptotic cell death in our study. These morphological and molecular hallmarks are consistent with the classical features of apoptosis reported in MCF-7 cells treated with vitamin D derivatives, including cell rounding, detachment, chromatin condensation, pyknotic nuclei, and nuclear matrix reorganization^46^.

Aspirin, widely recognized as an anti-thrombotic agent for the prevention of thromboembolic events, has also been shown to induce apoptosis in cancer cells. Several reports demonstrate that aspirin and other NSAIDs can trigger mitochondrial cytochrome c release^47,48^, leading to the activation of the caspase cascade^47^. Additionally, aspirin-mediated modulation of apoptotic regulators specifically, the upregulation of pro-apoptotic Bax and suppression of anti-apoptotic Bcl-2^49^ suggests a multifaceted mechanism underlying its potential anticancer properties. DNA fragmentation observed in the present study further supports aspirin-induced apoptosis.

Caspase-3, a well-established executioner caspase, functions downstream of the caspase-8-mediated death receptor pathway^50^. Upon activation, procaspase-8 undergoes proteolytic cleavage, subsequently activating effector caspases including caspase-3, -6, and − 7. This cascade orchestrates cellular disassembly through targeted proteolysis of structural and nuclear substrates, culminating in programmed cell death^51^.

According to previous studies^52^, apoptotic DNA fragmentation generates a characteristic “ladder pattern” of nucleosomal fragments of varying sizes, signifying internucleosomal cleavage and programmed cell death in hepatocytes. Bromadiolone, a potent second-generation anticoagulant, acts as a vitamin K antagonist through inhibition of vitamin K epoxide reductase, thereby impairing the synthesis of vitamin K-dependent proteins and indirectly contributing to DNA damage^53^. In the present study, rats exposed to aspirin or a combination of bromadiolone, ciprofloxacin, vitamin D, and cinnamon exhibited significant DNA strand breaks, as evidenced by elevated comet assay parameters, indicating enhanced genotoxic stress.

Activated protein C (APC), a vitamin K-dependent serine protease, has been shown to exert anti-apoptotic effects by transcriptionally repressing p53, restoring the Bax/Bcl-2 ratio, and attenuating caspase-3 activation in hypoxic human brain endothelial cells^54^. The observed increase in apoptosis following bromadiolone exposure may therefore result from APC inhibition, which disrupts these cytoprotective pathways and promotes caspase-mediated cell death. This mechanistic disruption likely explains the prolonged prothrombin time (PT) and bleeding tendencies observed in treated groups.

Our findings are consistent with earlier reports^55,56^ showing that anticoagulant rodenticides inhibit vitamin K epoxide reductase, preventing the regeneration of reduced vitamin K and thereby blocking the activation of clotting factors II, VII, IX, and X. This deficiency leads to impaired hemostasis and hepatocellular injury. Prothrombin, a key vitamin K-dependent zymogen, appears to be particularly vulnerable to such inhibition, as it plays a central role in both intrinsic and extrinsic coagulation cascades^56^.

Given the liver’s pivotal role in maintaining the equilibrium between thrombosis and hemorrhage, severe hepatic and renal lesions observed in bromadiolone–cinnamon-treated rats likely reflect cumulative oxidative and coagulopathic injury. The rapid onset of mortality in this group further supports this interpretation. Moreover, the elevated PT values observed may also indicate hepatocellular dysfunction or cirrhotic changes. Consistent with this, prior pharmacokinetic studies⁵^57^ have shown that coumarin derivatives such as 4-hydroxycoumarin, p-coumaric acid, and coumaric acid are rapidly absorbed, widely distributed, and accumulate preferentially in the liver and kidneys. These compounds may potentiate the anticoagulant and hepatotoxic effects of bromadiolone, collectively contributing to the observed physiological outcomes.

Abnormalities in serum marker enzymes indicate a disorder affecting the tissue responsible for maintaining the balance between protein synthesis and catabolism or mechanical loss, rather than a specific disease. In the current study, the combination of bromadiolone, ciprofloxacin, Vit. D, and cinnamon, or the addition of aspirin, resulted in increased LPO, ALT and AST. This may be attributed to bromadiolone toxicity, which damages hepatocyte cell membranes or increases cell membrane permeability. The release of these marker enzymes due to liver’s accumulation of ROS may account for the increase in lipid peroxidation^10^.

For 21 days, rats were fed bromadiolone along with other additional additives, which resulted in reduced antioxidant GSH levels, primarily due to the inhibition of vitamin K activity by ROS. The use of this chemical combination with bromadiolone in wild rats has not been previously reported.

The effects of adding aspirin or the combination of ciprofloxacin, Vit. D, cinnamon, and bromadiolone on blood coagulation are reflected in the hematological results. Our investigation confirmed that hemorrhage may lead to decreased hemoglobin levels and erythrocyte loss. May also result from their migration out of the bone marrow, as these outcomes stem from bromadiolone’s impact on bone marrow hematopoiesis^58^. These findings are consistent with human studies involving bromadiolone, which reported low levels of Hb and Hct^59^ The results of the present study demonstrate that exposure to acetylsalicylic acid (aspirin) in Rattus rattus (black rat), under both laboratory and field conditions, markedly increased mortality, hemorrhagic manifestations, and severe histopathological alterations in multiple organs, including the brain, heart, lungs, liver, kidneys, and ovaries. The observed pathological changes are consistent with aspirin’s known pharmacological mechanism as a potent inhibitor of platelet aggregation through irreversible acetylation of cyclooxygenase (COX) enzymes, thereby suppressing thromboxane A₂ synthesis. Consequently, the treated animals exhibited a progressive and significant prolongation of bleeding time compared with control groups^60^. These findings suggest that even at sublethal exposure levels, aspirin-containing baits can induce systemic coagulopathic and multi-organ toxic effects, reflecting a cumulative disruption of hemostatic balance and vascular integrity. Because of ciprofloxacin’s broad-spectrum effectiveness against both gram-positive and gram-negative organisms, it is frequently used as an anti-infective treatment for genitourinary tract infections. Despite being safe at therapeutic dosages, new research points to possible sever damage to testicular histology and function^61^. According to Akhtara, et al.^61^ and as reported by Ibrahim et al.^62^; It has been demonstrated that experimental fluoroquinolone administration in mice causes negative effects on testis and liver damage. In this instance, mice exposed to Ciprofloxacin for a prolonged period of time experience oxidative stress and ROS production, which damages membranes, raises the amount of MDA, a byproduct of lipid peroxidation, and significantly weakens the antioxidant defense system, as shown by decreased GSH and CAT activity.

Co-administration of Ciprofloxacin in many animal species inhibits CYP1A activity in hepatic microsomes, causing irreversible and cumulative toxicity with CYP1A and 3 A substrates as well as major drug-drug interactions (DDIs)^63^. The primary cause might emphasize the clinical symptoms of impairment linked to fluoroquinolones: Long-term oxidative stress breaks down mtDNA, and ROS can also cause mitochondrial malfunction and liver damage by forming cytochrome complexes that leak electrons permanently and lead to oxidative stress^63^ Singh et al.^64^, agreed with our results, as they showed that: the exposure to acetaminophen carbon tetrachloride and thioacetamide caused damage to the liver’s histoarchitecture, liver function, lipid peroxidation, glutathione, superoxide dismutase, catalase, and GSH cycle enzyme activities, as well as DNA fragmentation.

The histological results further support our biochemical findings, as the experimental rat liver exhibited substantial damage compared to control rats. Rats in the cinnamon group experienced more severe damage than those in other groups, primarily due to the multiple harmful effects of cinnamon (like ROS and lipid peroxidation) when combined with bromadiolone in liver tissues. The histopathology slides were independently examined and scored by two authors of the current manuscript separately, then their results were compared and alignments were carried out. Similar findings were reported by other authors following ciprofloxacin administration^65^, who observed that the drug induced hepatocyte inflammation and necrosis, along with altered hepatic histoarchitecture, including apoptosis, congestion, and vacuolar degeneration.

## Conclusion

The findings of this study suggest that bromadiolone, when combined with ciprofloxacin, vitamin D, cinnamon, or aspirin, may contribute to enhanced apoptotic responses in liver tissue, as indicated by modulation of p53, p21, cleaved caspase-3, lipid peroxidation, DNA damage, and prothrombin time. Among these additives, cinnamon appeared to show potential in augmenting the efficacy of bromadiolone, which could allow for the use of reduced quantities of the rodenticide. Such an approach might help to minimize environmental burden and lower the risk to non-target organisms. Nevertheless, further investigations with larger sample sizes, additional molecular endpoints, and field-based evaluations are warranted before drawing definitive conclusions about the applicability of these combinations in rodent control strategies.

## Supplementary Information

Below is the link to the electronic supplementary material.

Supplementary Material 1

Supplementary Material 2

## Data Availability

All data generated or analyzed during this study are included in this published article [and its supplementary information files].

## Abbreviations

B: bromadiolone
Vit. D: vitamin D
A: aspirin
Cin: cinnamon
GSH: reduced glutathione
AO: acridine orange
ALT: alanine aminotransferase
AST: aspartate aminotransferase
LPO: lipid peroxidation
