# Supplementary material for: Evaluation of bromadiolone combined with ciprofloxacin, vitamin D, aspirin, and cinnamon as an apoptosis-mediated rodenticide strategy
Source: Sci Rep. 2025 Dec 8;15:43385. doi: 10.1038/s41598-025-28468-7 (PMC12689647; doi:10.1038/s41598-025-28468-7)
Supplement: Supplementary file 1 — Supplementary Material 1 [file 41598_2025_28468_MOESM1_ESM.pdf]

Fig. 1

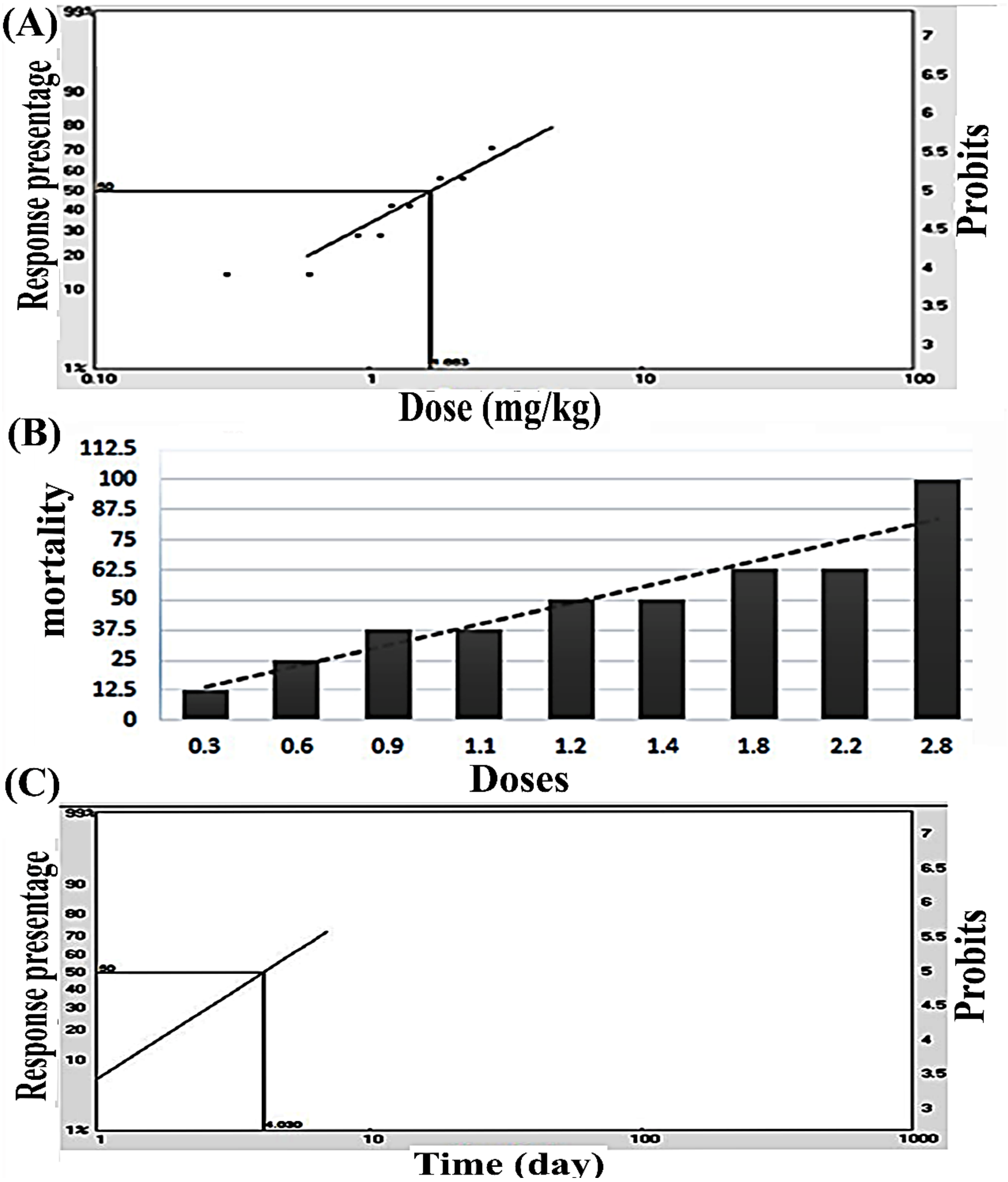

(A)
Response presentage
Probits
Dose (mg/kg)
(B)
mortality
Doses
0.3
0.6
0.9
1.1
1.2
1.4
1.8
2.2
2.8
(C)
Response presentage
Probits
Time (day)

Fig. 2

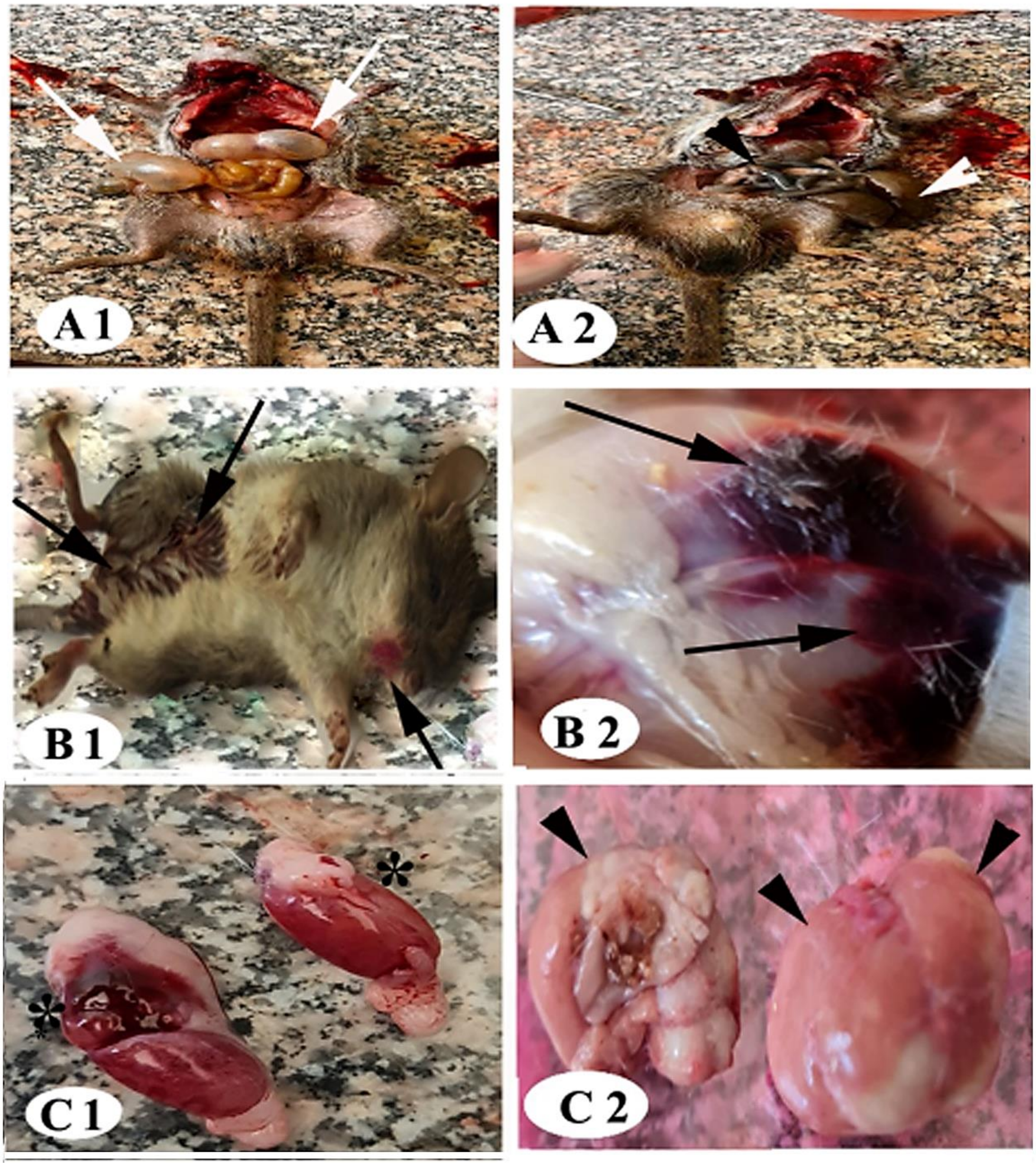

Fig. 3

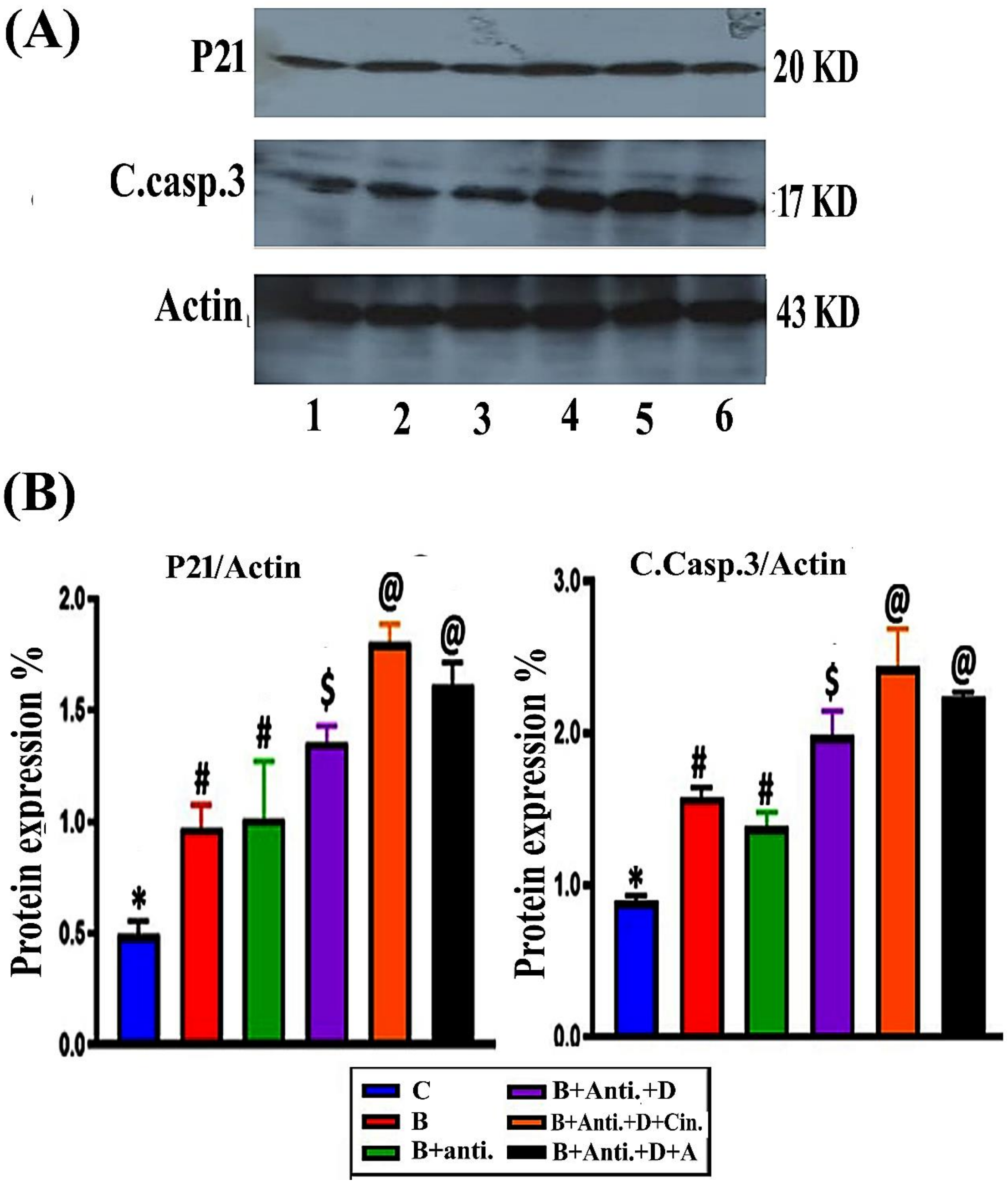

(A)
P21
20 KD
C.casp.3
17 KD
Actin
43 KD
1
2
3
4
5
6
(B)
P21/Actin
C.Casp.3/Actin
Protein expression %
C
B
B+anti.
B+Anti.+D
B+Anti.+D+Cin.
B+Anti.+D+A

Fig. 4

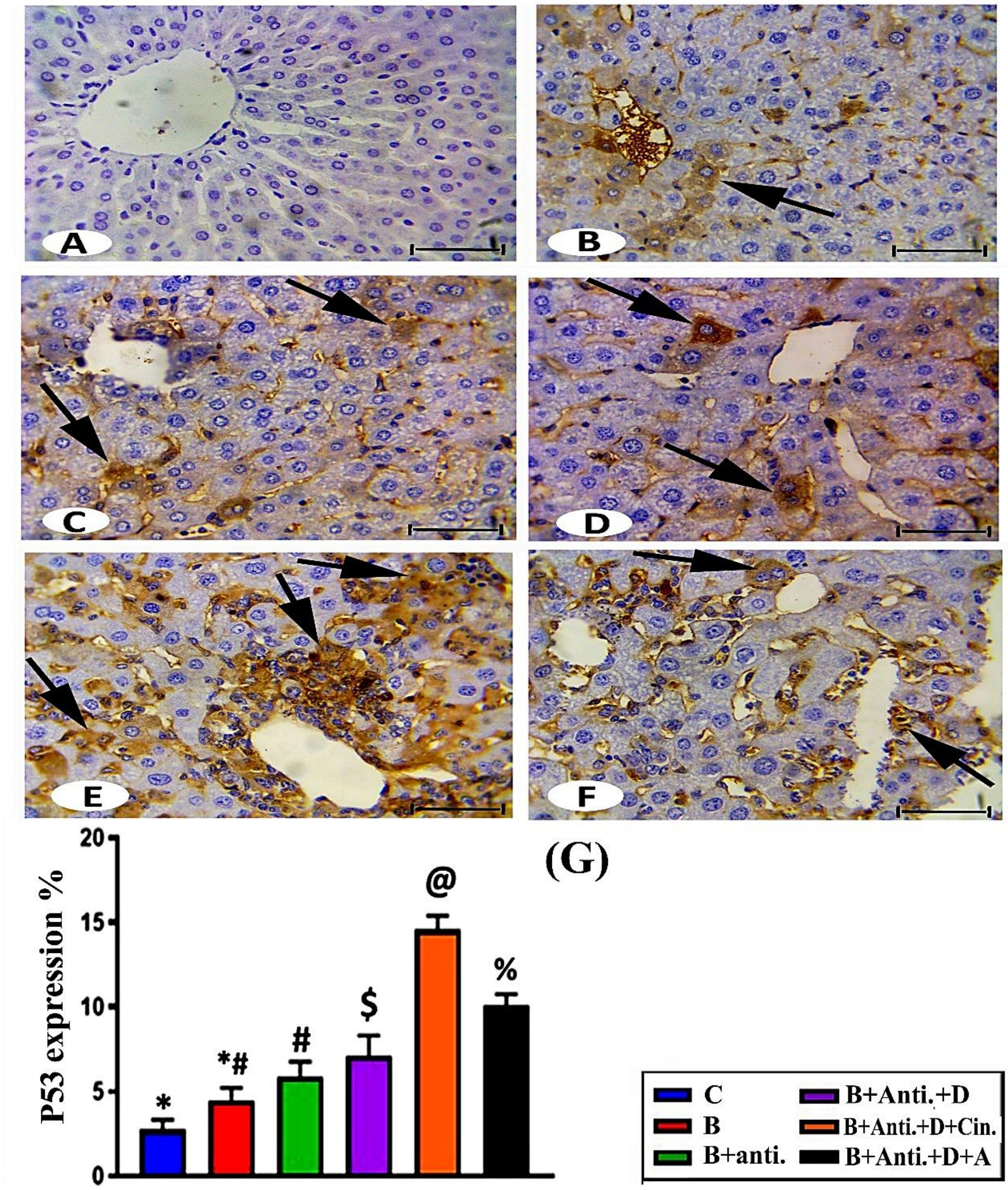
A
B
C
D
E
F
(G)
P53 expression %
20
15
10
5
0
*
*#
#
$
@
%
C
B
B+anti.
B+Anti.+D
B+Anti.+D+Cin.
B+Anti.+D+A

Fig. 5

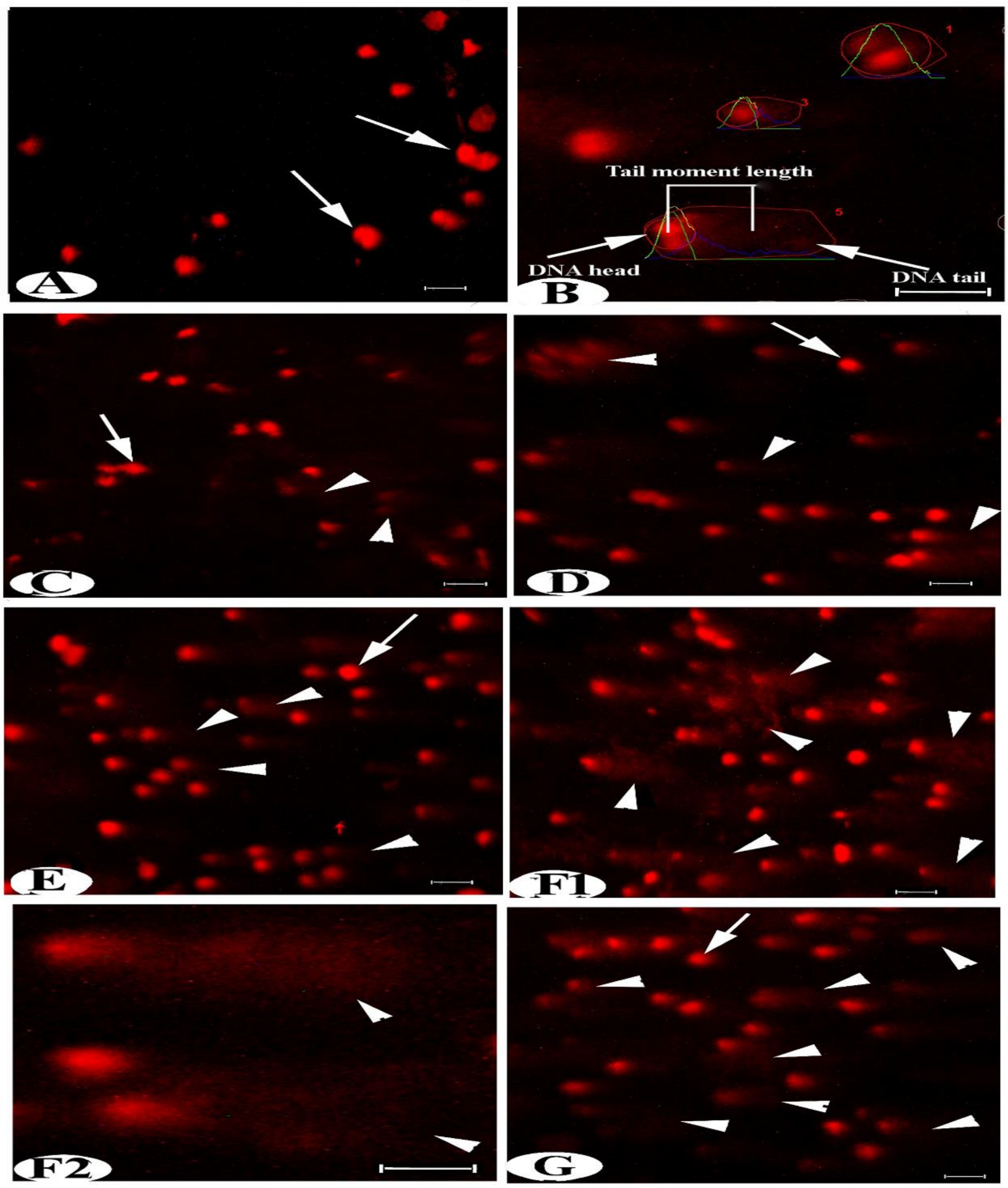

Fig. 6

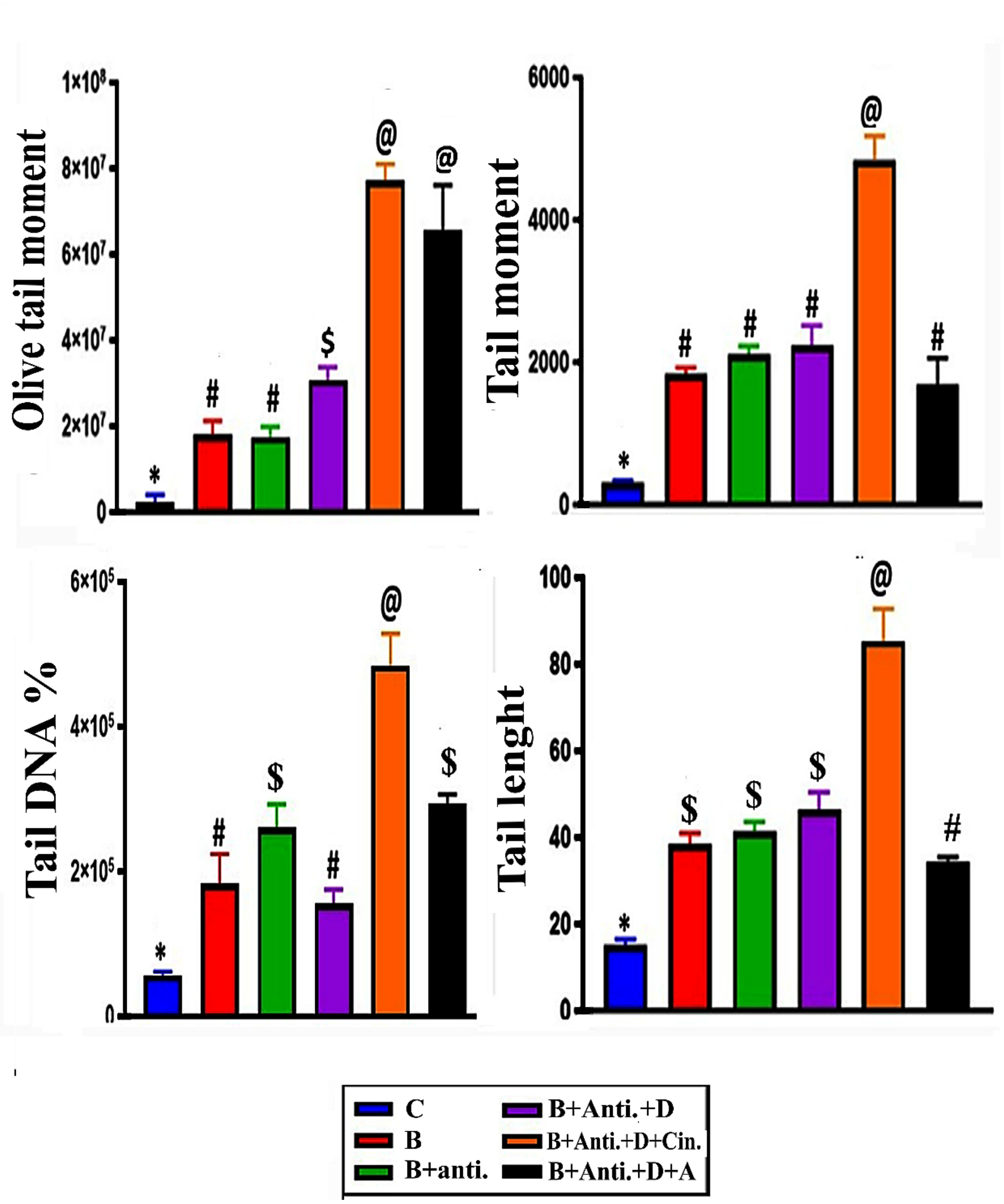

Olive tail moment
1×10⁸
8×10⁷
6×10⁷
4×10⁷
2×10⁷
0
Tail moment
6000
4000
2000
0
Tail DNA %
6×10⁵
4×10⁵
2×10⁵
0
Tail lenght
100
80
60
40
20
0
C
B
B+anti.
B+Anti.+D
B+Anti.+D+Cin.
B+Anti.+D+A

Fig.7

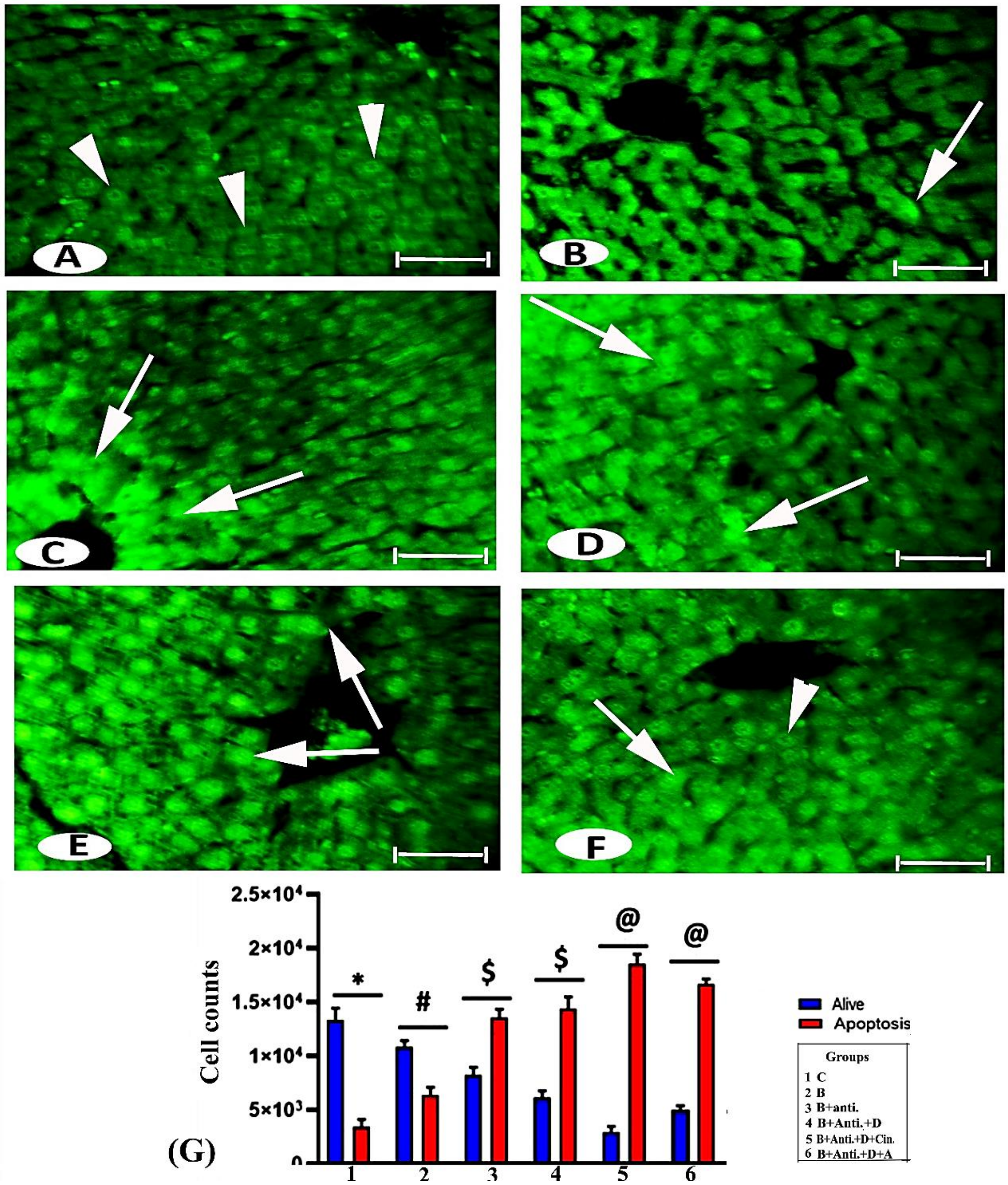
A
B
C
D
E
F
2.5×10⁴
2×10⁴
1.5×10⁴
1×10⁴
5×10³
0
Cell counts
(G)
1
2
3
4
5
6
*
#
$
$
@
@
Alive
Apoptosis
Groups
1 C
2 B
3 B+anti.
4 B+Anti.+D
5 B+Anti.+D+Cin.
6 B+Anti.+D+A

Fig. 8

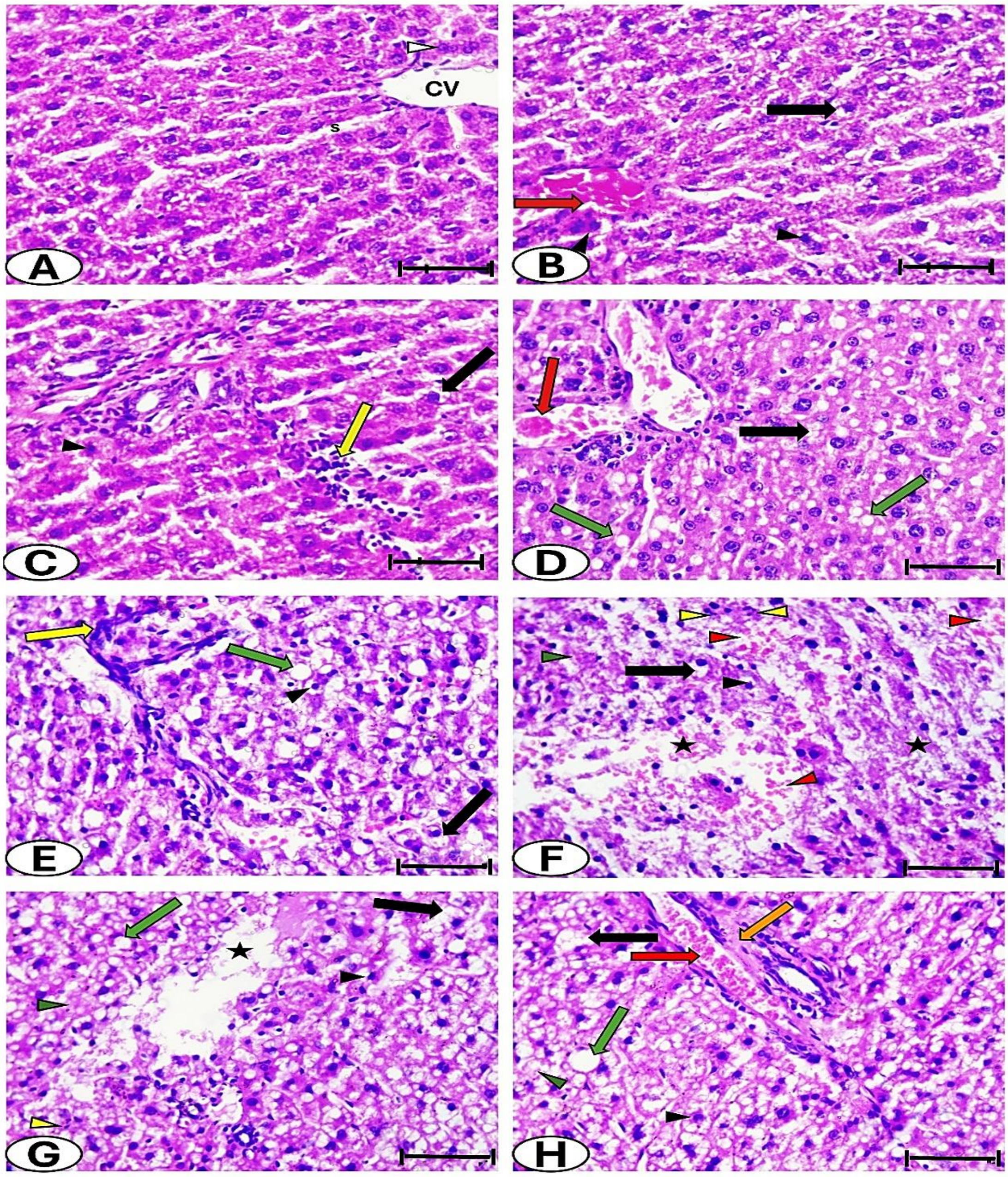

Table (1)

Dose	Dose*10	Log (Dose*10)	Treated	Dead	Mortality%	Observed response %	Linear response %	Linear probit
**0.3**	3	0.4771	8	1	12.5	14.286	8.15909	3.6051
**0.6**	6	0.7782	8	2	25	14.286	20.3258	4.1699
**0.9**	9	0.9542	8	3	37.5	28.571	30.855	4.5
**1.1**	11	1.0414	8	3	37.5	28.571	36.8278	4.6635
**1.2**	12	1.0792	8	4	50	42.857	39.5276	4.7344
**1.4**	14	1.1461	8	4	50	42.857	44.4304	4.8599
**1.8**	18	1.2553	8	5	62.5	57.143	52.578	5.0647
**2.2**	22	1.3424	8	5	62.5	57.143	59.022	5.2281
**2.8**	28	1.4472	8	8	100	71.429	66.4427	5.4246

Table (2):

LD	Dose (mg/kg)
25	0.7264
50	1.6626
75	3.8056
90	8.0187
95	12.5257
99	28.9136

Table (3)

Time	Time* 1	Log (Time*1)	Treated	Observed response %	Linear response %	Linear probit
1	1	0	8	12.5	6.20873	3.4617
2	2	0.301	8	12.5	21.9652	4.2266
3	3	0.4771	8	37.5	37.2284	4.6741
4	4	0.6021	8	50	49.6729	4.9918
5	5	0.699	8	50	59.4089	5.2381
6	6	0.7782	8	62.5	66.9788	5.4393
7	7	0.8451	8	87.5	72.8843	5.6093

Table (4)

LT	Time (day)	Lower limit day	Upper limit day
**25**	2.187	0.822	3.091
**50**	4.03	2.771	6.273
**75**	7.426	5.129	23.18
**90**	12.87	7.592	88.39
**95**	17.89	9.453	200
**99**	33.17	14.11	934.7

Table (5)

	Male Rattus norvegicus										
	**Control**	**B**		**(B + anti)**		**(B +anti+ vit. D)**		**(B + anti + vit. D+C)**		**(B + anti + vit. D+A)**	
	**Mean±SE**	**Mean±SE**	**% of change**	**Mean±SE**	**% of change**	**Mean±SE**	**% of change**	**Mean±SE**	**% of change**	**Mean±SE**	**% of change**
**AST (U/L) serum**	92.37 ± 5.279 *	130.8 ± 8.149 #	41.55 % ↑	165.1 ± 5.088 $	78.71 % ↑	186.5 ± 3.686 $%	101.86% ↑	248.0 ± 4.954 @	168.56% ↑	210.0 ± 9.578 &	127.31% ↑
**ALT (U/L) serum**	184.8 ± 4.632 *	225.5 ± 1.679 *	22.02% ↑	215.3 ± 1.715 *	16.51% ↑	251.7 ± 7.382 *	36.22 % ↑	364.2 ± 38.45 @	97.08% ↑	385.1 ± 18.23 @	108.39% ↑
**LPO (nmol/ mg protein)**	1.458 ± 0.1402 *	1.660 ± 0.1817*	13.69 % ↑	2.530 ± 0.2937#	73.29 % ↑	2.984 ± 0.2613 #	104.79 % ↑	5.313 ±0.3714@	264.38% ↑	3.903 ± 0.2003 $	167.12% ↑
**GSH (ng/mg protein)**	10.42 ± 0.4500 *	6.953 ± 0.1538#	33.21 % ↓	5.893 ± 0.2378 #	43.38 % ↓	5.177 ± 0.7614#	50.29% ↓	3.823 ±0.4656@	63.24 % ↓	5.373 ± 0.3210 #	48.37 % ↓
**Ca2+ (mg/dl) serum**	4.640 ± 0.2943 *	13.15 ±0.9675 #	183.41% ↑	11.18 ± 0.8499 #	140.95% ↑	17.12 ± 1.965 $	268.97% ↑	23.32 ± 2.970 @	402.59% ↑	18.76 ± 2.705 $	304.31% ↑
**RBCs (M/µl) serum**	11.08 ± 0.1956 *	8.240 ± 0.5015 #	25.63% ↓	8.192 ± 0.2049 #	26.08% ↓	6.098 ± 0.9436 $	44.95% ↓	4.396 ± 0.8644 @	60.29% ↓	5.054 ± 0.9225 $	54.33% ↓
**Hgb (g/dl) serum**	11.92 ± 0.3813*	11.4 ± 0.2358*	7.38% ↓	10.02 ± 0.8279 #	15.94% ↓	7.980 ± 1.403 $	33.05% ↓	6.160 ± 1.145 @	48.32% ↓	8.260 ± 1.047 $	30.70% ↓
**PT (Sec.) serum**	21.25 ± 2.212 *	37.43 ± 1.329 *	76.14% ↑	93.18± 3.277 $	338.49% ↑	144.2 ± 7.471 #	578.82% ↑	258.6 ± 8.295 @	1116.71% ↑	243.8 ± 9.290 @	1047.06% ↑

Table (6)

Lesions / Groups	Control	B	B+Anti	B+Anti+D	B+Anti+D+C	B+Anti+D+[illegible]
**Loss of liver architecture**	-	-	+	++	+++	+++
**Features of necrosis:**						
- **Pyknosis**	+	++	++	+++	+++	+++
- **Karyorrhexis**	-	+	+	+	+	++
- **Karyolysis**	-	+	++	+	++	++
**Vacuolated cytoplasm**	+	++	+++	+++	+++	+++
**Fatty deposition**	-	-	++	+++	+++	++
**Congested blood vessels**	-	++	++	++	+++	++
**Dilated blood sinusoids**	-	+	+	+	++	++
**Hemorrhage**	-	-	+	++	++	+++
**Thickening of blood vessels**	-	-	-	+	++	+
**Cellular infiltration**	-	++	++	+++	+++	++
**Necrotic areas**	-	+	+	+	+++	+++
