# Supplementary figures and images for: Evaluation of bromadiolone combined with ciprofloxacin, vitamin D, aspirin, and cinnamon as an apoptosis-mediated rodenticide strategy
Source: Sci Rep. 2025 Dec 8;15:43385. doi: 10.1038/s41598-025-28468-7 (PMC12689647; doi:10.1038/s41598-025-28468-7)

actin

casp.3

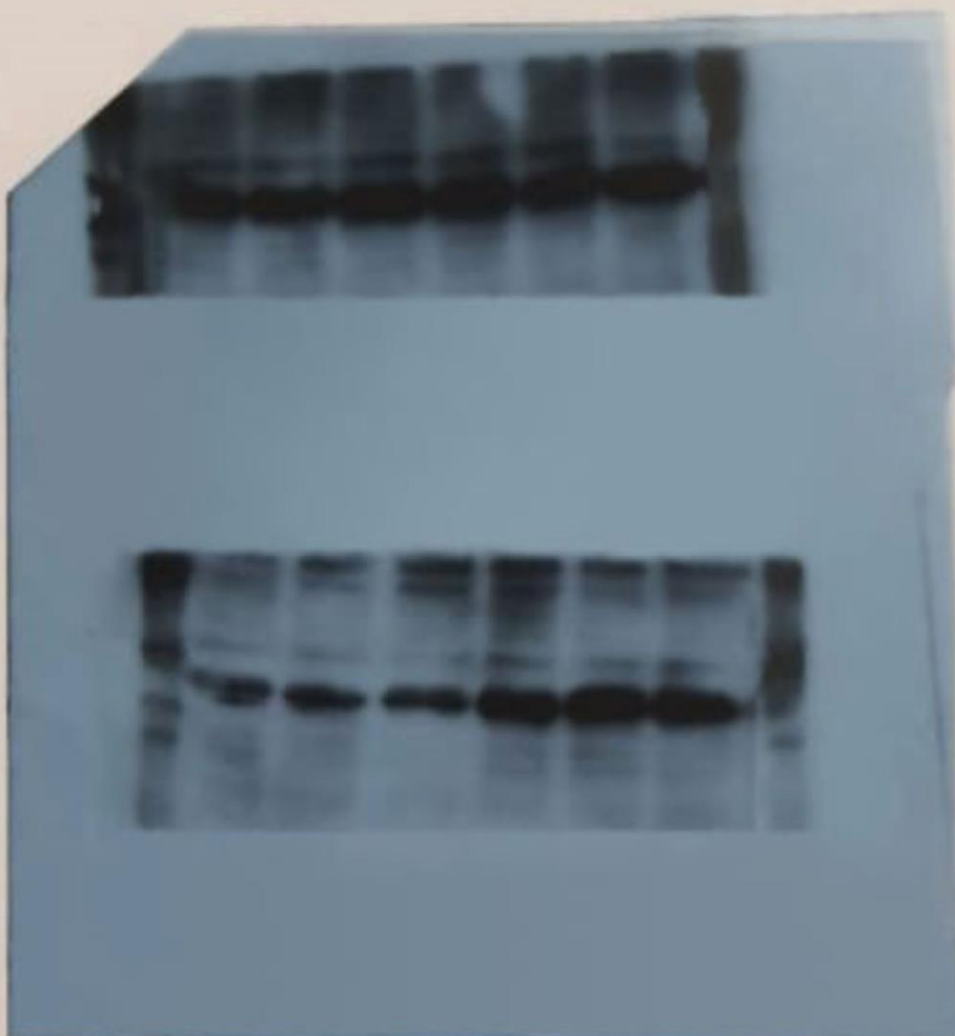

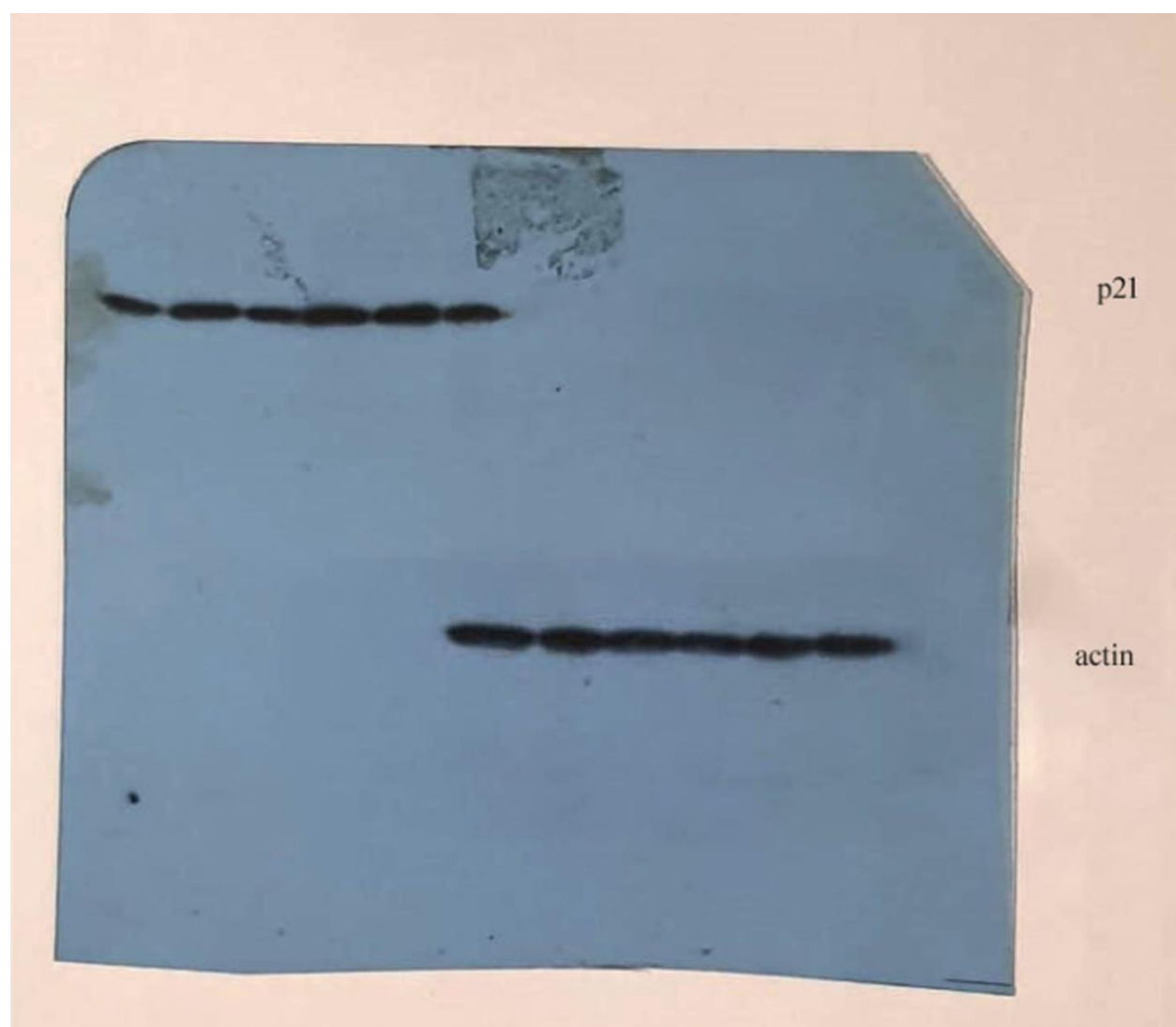

Supplement: Supplementary file 2 — Supplementary Material 2 [file 41598_2025_28468_MOESM2_ESM.pdf]
